# Lung macrophage scavenger receptor SR-A6 (MARCO) is an adenovirus type-specific virus entry receptor

**DOI:** 10.1371/journal.ppat.1006914

**Published:** 2018-03-09

**Authors:** Nicole Stichling, Maarit Suomalainen, Justin W. Flatt, Markus Schmid, Martin Pacesa, Silvio Hemmi, Wolfgang Jungraithmayr, Mareike D. Maler, Marina A. Freudenberg, Andreas Plückthun, Tobias May, Mario Köster, György Fejer, Urs F. Greber

**Affiliations:** 1 Department of Molecular Life Sciences, University of Zurich, Zurich, Switzerland; 2 Molecular Life Sciences Graduate School, ETH and University of Zurich, Switzerland; 3 Department of Biochemistry, University of Zurich, Zurich, Switzerland; 4 University Hospital Zurich, Institute of Thorax Surgery, Zurich, Switzerland; 5 present address: Department of Thoracic Surgery, Medical University Brandenburg, Neuruppin, Germany; 6 Max-Planck Institute of Immunobiology and Epigenetics, Freiburg, Germany; 7 Allergy Research Group, Department of Dermatology, Medical Center—University of Freiburg, Faculty of Medicine, University of Freiburg, Freiburg, Germany; 8 Faculty of Biology, University of Freiburg, Freiburg, Germany; 9 BIOSS Centre for Biological Signalling Studies, Albert-Ludwigs-Universität, Freiburg, Germany; 10 Department of Pneumology, Medical Center–University of Freiburg and Faculty of Medicine, University of Freiburg, Freiburg, Germany; 11 Inscreenex GmbH, Inhoffenstr. Brunswick, Germany; 12 Helmholtz-Zentrum für Infektionsforschung GmbH, Braunschweig, Germany; 13 School of Biomedical and Healthcare Sciences, Peninsula Schools of Medicine and Dentistry, University of Plymouth, Plymouth, United Kingdom; Penn State University School of Medicine, UNITED STATES

## Abstract

Macrophages are a diverse group of phagocytic cells acting in host protection against stress, injury, and pathogens. Here, we show that the scavenger receptor SR-A6 is an entry receptor for human adenoviruses in murine alveolar macrophage-like MPI cells, and important for production of type I interferon. Scavenger receptors contribute to the clearance of endogenous proteins, lipoproteins and pathogens. Knockout of SR-A6 in MPI cells, anti-SR-A6 antibody or the soluble extracellular SR-A6 domain reduced adenovirus type-C5 (HAdV-C5) binding and transduction. Expression of murine SR-A6, and to a lower extent human SR-A6 boosted virion binding to human cells and transduction. Virion clustering by soluble SR-A6 and proximity localization with SR-A6 on MPI cells suggested direct adenovirus interaction with SR-A6. Deletion of the negatively charged hypervariable region 1 (HVR1) of hexon reduced HAdV-C5 binding and transduction, implying that the viral ligand for SR-A6 is hexon. SR-A6 facilitated macrophage entry of HAdV-B35 and HAdV-D26, two important vectors for transduction of hematopoietic cells and human vaccination. The study highlights the importance of scavenger receptors in innate immunity against human viruses.

## Author summary

Macrophages are a diverse group of phagocytic cells acting in host protection against stress, injury, and pathogens. They phenotypically and functionally adapt to their local environment, for example, peritoneal macrophages are distinct from brain-resident microglia, from liver-resident Kupffer cells or lung macrophages in the lung. Airway macrophages are among the first cells to encounter human respiratory viruses, such as adenoviruses. They release pro-inflammatory cytokines, kill pathogens, present antigens, and restore tissues. Yet, interactions of viruses with lung macrophages are poorly understood, and it is unclear, how they lead to infection or virus clearance. Here we identified the murine scavenger receptor SR-A6 as a receptor for a subset of human adenoviruses on alveolar macrophage-like cells, so-called MPI cells. Scavenger receptors comprise a large family of trans-membrane proteins, and contribute to the clearance of endogenous proteins, lipoproteins and pathogens. In a series of robust experimentation, we show that adenoviruses use SR-A6 as an entry receptor for infection of MPI cells, and production of type I interferon. MPI cells are non-transformed, self-renewing macrophages derived from fetal murine liver, and closely resemble adult alveolar macrophages. The results demonstrate that SR-A6 binds virions on the surface of alveolar macrophage-like cells, and leads to infection.

## Introduction

Macrophages are a diverse population of innate immune cells. They function as a first-line defense against pathogens, including viruses. Most tissues contain macrophages, and these macrophages differ by origin, repertoire of surface receptors and transcriptional regulation of many genes [1]. Macrophages are seeded into their tissue-resident environment during embryogenesis or differentiate from monocytes and develop plasticity and function in response to signals from their microenvironment [reviewed in 2, 3, 4]. Macrophages contain a broad array of surface receptors for recognition and engulfment of pathogens, including fungi, bacteria and viruses [5–8]. Different studies have shown that murine cytomegalovirus (MCMV), vaccinia virus (VacV) or human adenovirus (HAdV) infections induce the production of type I interferon (IFN) [9, 10]. A key element of how macrophages function and elicit inflammatory signaling is that they bind to pathogens by their cell surface receptors.

An important and widely expressed class of macrophage surface receptors are scavenger receptors (SRs). SRs constitute a large family of structurally diverse cell surface receptors, and span the membrane once or twice. SRs interact with and mediate uptake of a wide range of ligands, such as modified and non-modified self-molecules, non-opsonized particles and microbial ligands [11, 12]. On macrophages, SRs recognize a broad range of ligands, and are important for the clearance of foreign particulate material [12–14]. For example, human and murine SR-A6 have been implicated in infection of epithelial cells with herpes simplex virus type 1 (HSV-1), and SR-A1 and SR-F1/2 (SREC-1) are surface receptor candidates for HAdV-C5 on Kupffer cells and liver sinusoidal endothelial cells [15–18].

A virus receptor on the cell surface is a gatekeeper of viral invasion into the host, triggering virus production or destruction. The receptor makes direct contact with the virus particle, the virion. The outer structure of a virion consists of repetitively arranged proteins, and in case of enveloped viruses also lipids and sugars. This enables the engagement of multiple receptor molecules to a virion, the recruitment of host signaling molecules, and leads to the formation of an endocytic pit, and virion uptake into an endosome [19–22]. Receptor binding to virions also initiates structural changes in the particle which, together with additional cues from the cell, enable the virus to penetrate into the cytosol [22–24]. For example, the coxsackie adenovirus receptor (CAR) mediates initial attachment of species A and C HAdVs to non-immune cells [25–27], distinct from the membrane cofactor protein CD46, or desmoglein-2, which are the main receptors for species D and B viruses, respectively [28–30]. CAR engages retrograde acto-myosin mediated transport and exerts mechanical stress on HAdV-C5 against the holding force of the secondary integrin receptors, which leads to the exposure of the membrane lytic protein of the virion [31, 32]. CAR, CD46 and DSG-2 interactions with HAdV are mediated by the globular head domain (knob) of the fiber which protrudes from the vertex of the icosahedral virion [33]. Yet, CAR and DSG-2 are not expressed on macrophages, and although CD46 is a ubiquitously expressed protein in humans, its expression in mouse is restricted to testis [34].

HAdVs are non-enveloped, double stranded DNA viruses. They replicate in the host cell nucleus. Their outer surface is composed of three main proteins, hexon, penton base and fiber. Penton base and fiber localize to the capsid vertices, whereas the structural framework of the capsid is formed by hexons [35, 36]. HAdVs are classified into seven species A to G with more than 50 serotypes, and nearly 70 bioinformatically defined types [37]. HAdV infections in immuno-competent hosts manifest with mild respiratory (HAdV-B, -C, -E), ocular (HAdV-B, -D, -F) or gastrointestinal infections (HAdV-F). The widespread application of HAdV-based vectors in gene therapy, cancer gene therapy and vaccination enhances the medical importance of these viruses [38–40].

HAdV-C5 based toxicity is strongly linked to innate immune responses against the virions [10, 41–43]. Macrophages are central actors in these responses. For example, intravenous administration of HAdV-C5-derived vectors into mice leads to rapid capture of the vector particles by the liver resident macrophages, the Kupffer cells, as well as by the spleen marginal zone macrophages and myeloid dendritic cells, and these cells contribute to the vector-induced early production of proinflammatory cytokines, such as type I IFNs, interleukin-1α (IL-1α), interleukin-6 (IL-6) and tumor necrosis factor (TNF) [10, 44–46]. Furthermore, lung macrophages capture HAdV-C5-derived vectors administered via the respiratory tract and respond with a rapid induction of TNF and IL-6 [47, 48]. Finally, mouse alveolar macrophage-like MPI cells produce robust levels of cytokines upon inoculation with early gene expression defective HAdV-C5_dE1_GFP [49]. These acute phase macrophage responses are directly triggered by the incoming virions, and critically require interaction of the incoming viral genome with the cytoplasmic DNA sensor cyclic GMP-AMP synthase (cGAS) and/or the endosomal DNA sensor Toll-like receptor 9 (TLR9) [44, 50–53]. However, virus-host interactions that mediate attachment and uptake of adenoviruses into macrophages are incompletely understood.

In the present study, we made use of the recently developed mouse alveolar macrophage-like MPI (MPI-2) cells derived from C57BL/6 (BL6) mice [49] and identified the scavenger receptor SR-A6 (MARCO; macrophage receptor with collagenous structure) as a receptor for HAdV-C5. We show that knockdown or knockout of the SR-A6 gene, anti-SR-A6 antibody or soluble extracellular domain of SR-A6 inhibited binding of HAdV-C5 to MPI-2 cells and virus-mediated gene transduction. EM studies indicated that the soluble SR-A6 caused clustering of HAdV-C5 particles, implying that SR-A6 directly interacts with the virion. In support of direct SR-A6 interaction with HAdV-C5, proximity ligation assays of HAdV-C5 on SR-A6 positive cells demonstrated that cell surface-bound virions were in close proximity to SR-A6. Genetic swapping of the negatively charged hypervariable region 1 (HVR1) of HAdV-C5 hexon with the short HVR1 of HAdV-A31 reduced virus binding and virus-mediated gene transduction of MPI-2 cells. HAdV-C5 binding to SR-A6 initiated an entry pathway leading to efficient nuclear targeting of the incoming virus, and gene transduction. SR-A6 not only supported HAdV-C5 entry and transduction, but also enabled HAdV-C2, HAdV-B35 and HAdV-D26 entry into macrophages. Although the human orthologue of SR-A6 enhanced virion binding to and transduction of receptor-negative cells, it was a weaker receptor for HAdV-C5 than mSR-A6.

## Results

### SR-A6 is required for HAdV-C5 infection of murine alveolar macrophage-like MPI-2 cells

Limited supply of primary tissue macrophages and the sparseness of tissue culture models for macrophages has hampered characterization of host molecules that facilitate uptake of HAdVs into tissue macrophages. MPI (Max Planck Institute)-2 cells are non-transformed, self-renewing alveolar macrophage-like cells derived from mouse fetal liver [49], the organ that seeds the precursor cells that later develop into lung macrophages [54]. Alveolar macrophages are operationally defined here as those macrophages that are recovered from lung lavages. In contrast to bone marrow-derived macrophages (BMM), robust pro-inflammatory responses to HAdV-C are elicited in MPI-2 cells [49]. Likewise, human BMMs are not productively infected with HAdV species C although they respond to virions by an inflammatory response [55]. This suggests that MPI-2 cells are more susceptible to HAdV-C infection than BMMs. Comparison of BMM and MPI-2 transcriptomes indicated that the scavenger receptor SR-A6 (MARCO) mRNAs were significantly more abundant in MPI-2 cells than in BMMs, whereas other scavenger receptors displayed similar transcript levels in both cell types [49, 53].

**Fig 1:**
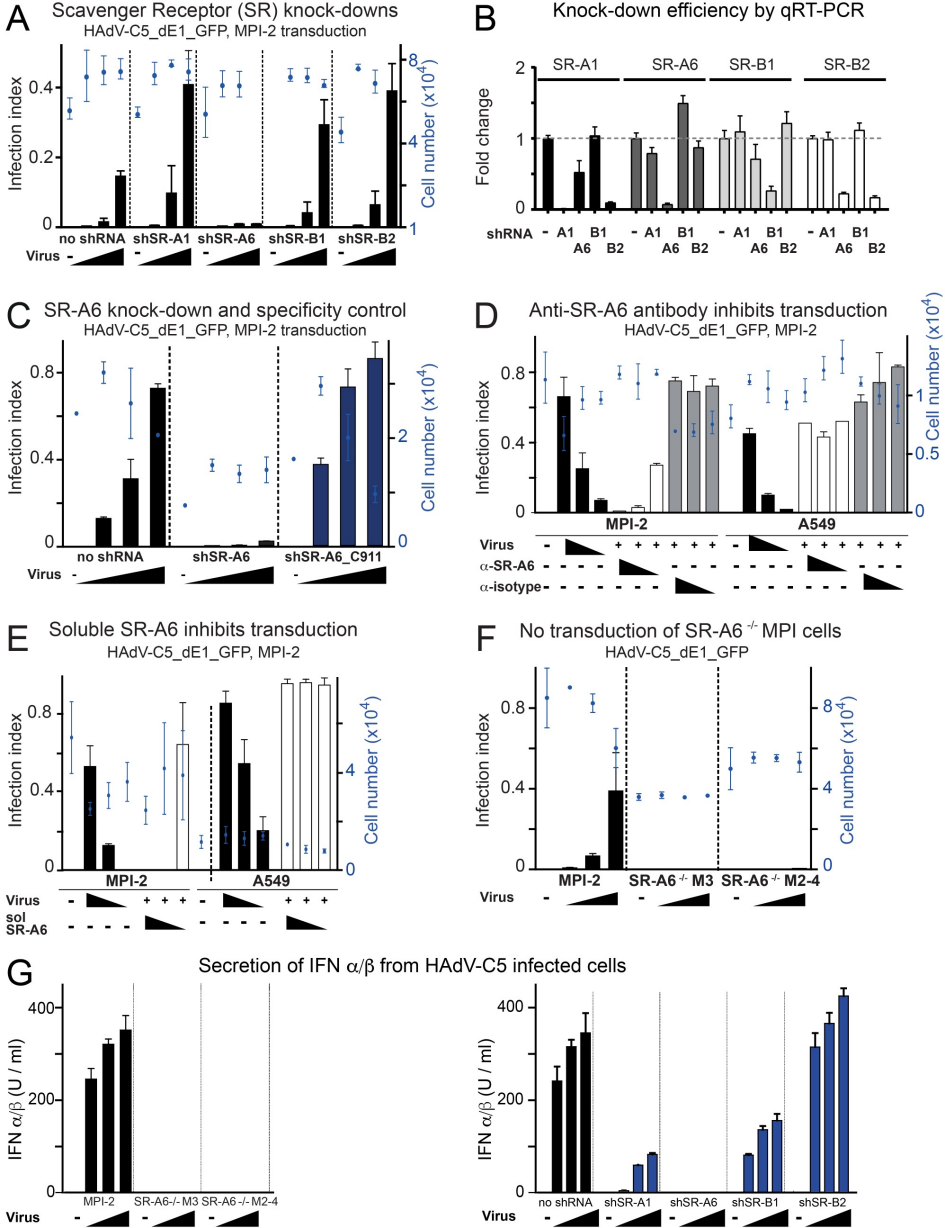
SR-A6 is required for HAdV-C5 infection of murine alveolar macrophage-like MPI-2 cells. A) Transduction assay with HAdV-C5_dE1_GFP in MPI-2 cells expressing shRNAs against the scavenger receptors SR-A1, SR-A6, SR-B1 and SR-B2. Three different input amounts of HAdV-C5_dE1_GFP (~9765–39000 virus particles per cell) were used for the transduction. Transduction efficiency was scored at 20 h pi by measuring GFP signal intensity over nucleus, and is expressed as infection index (number of GFP positive nuclei/total number of nuclei). The values represent mean values from three technical replicates ± standard deviations. B) qRT-PCR control of knockdown efficiencies in shRNA-expressing cells. Transcripts analyzed are indicated on the upper part of the panel, and the annotation on x-axis indicates shRNA-expressing cell lysates used for analyses. The transcript levels are normalized to the wild type MPI-2 cells by three or two house-keeping genes. C) Transduction assay with HAdV-C5_dE1_GFP on MPI-2 cells expressing shRNA against SR-A6 or a control, non-targeting SR-A6_C911 shRNA. D) Transduction assay with HAdV-C5_dE1_GFP on MPI-2 and A549 cells after pre-incubation of cells with three different concentrations of anti-SR-A6 antibody ED31 or an isotype-matched control antibody. E) Transduction assay with HAdV-C5_dE1_GFP on MPI-2 and A549 cells after pre-incubation of virus with three different amounts of soluble mouse SR-A6. Input virus ~16700 virus particles per cell with the soluble SR-A6. F) Transduction assay with HAdV-C5_dE1_GFP on wild type MPI-2 and two independent SR-A6^-/-^ MPI-cell lines. G) Secretion of IFN α/β from wild type, SR-A6 knock-out or scavenger receptor shRNA-expressing MPI-2 cells upon HAdV-C5 infection. Culture medium was collected from virus-infected cells 24h pi and titrated on a reporter cell line expressing the Firefly luciferase gene under the control of an IFN-inducible Mx2 promoter.

This prompted us to investigate whether SR-A6 could act as a receptor for HAdV-C5 in MPI-2 cells. As shown in Fig 1A, shRNA-mediated knockdown of SR-A6 in MPI-2 cells significantly reduced GFP transgene expression from the non-replicating HAdV-C5_dE1_GFP vector, whereas no reduction in infection efficiency was observed upon knockdown of other scavenger receptors expressed in MPI-2 cells, i.e. SR-A1 (Msr1), SR-B1 (SCAR-B1) or SR-B2 (CD36). Efficient knockdown levels were confirmed by qRT-PCR (Fig 1B). Of note, SR-A6 shRNA reduced SR-A1 and SR-B2 transcripts, but since both SR-A1 or SR-B2 shRNAs actually boosted HAdV-C5_dE1_GFP infection despite efficient knockdown of the respective transcripts, SR-A1 and SR-B2 are unlikely to be connected to the SR-A6 shRNA infection phenotype. To exclude seed-mediated off-target effects for the SR-A6 shRNA phenotype, we employed the so-called C911 control [56]. In this control, the shRNA bases 9 to 11 are replaced with complementary bases. As a result, on-target effects cannot be observed anymore, but off-target effects owing to sequences akin to microRNA seed sequences persist. In virus transduction assays, MPI-2 cells expressing the SR-A6_C911 shRNA behaved like the parental cells and displayed high GFP expression upon HAdV-C5_dE1_GFP infection (Fig 1C), thus confirming the on-target effect of the SR-A6 shRNA. Preincubation of MPI-2 cells with a blocking antibody against mouse SR-A6 (ED31) significantly reduced HAdV-C5_dE1_GFP-mediated transgene expression in a dose-dependent manner, whereas equivalent amounts of an isotype-matched negative control antibody had essentially no effect (Fig 1D). In contrast, in A549 cells, which are epithelial lung carcinoma cells and express CAR, in contrast to MPI-2 cells which are devoid of CAR expression [49], preincubation of A549 cells with the ED31 antibody did not reduce HAdV-C5_dE1_GFP infection (Fig 1D). Furthermore, preincubation of virus with soluble form of mouse SR-A6 blocked infection in a dose-dependent manner, whereas soluble SR-A6 had essentially no effect on virus-mediated GFP expression in A549 cells (Fig 1E). Taken together, these results suggest that SR-A6 is required for efficient HAdV-C5 infection of the MPI-2 cells.

To strengthen this notion, we used two SR-A6^-/-^ MPI cell lines (named M3 and M2-4) from fetal liver of SR-A6 knock-out mice. These cells did not express SR-A6 at their surface (S1A Fig.) and SR-A6 mRNAs were not detectable in these cells, whereas transcripts for SR-B1 were slightly reduced or, in the case of SR-A1 and SR-B2, actually increased (S1B Fig.). Data in Fig 1F demonstrate that the HAdV-C5_dE1_GFP transduction of M3 and M2-4 cells is significantly less efficient than the transduction of wild type MPI-2 cells, in agreement with [53].

MPI-2 cells have been previously described to respond to HAdV-C5 infection by secreting IL-6 and IL-1 [49]. We monitored interferon (IFN) α/β response from wild type and SR-A6-deficient MPI cells upon HAdV-C5 infection by titrating media from virus-infected cells in MEF-Mx2-luc-BKO reporter cell line. This cell line expresses firefly luciferase under the control of IFNα/β-inducible Mx2 promoter. As shown in Fig 1G, IFNα/β was released from infected MPI-2 cells, but not from M3 and M2-4 SR-A6 knockout cells. Similarly, HAdV-C5 did not evoke IFNα/β secretion from MPI-2 cells expressing SR-A6 shRNAs, but the virus did induce efficient IFNα/β response from cells expressing the control non-targeting SR-A6_C911 shRNA (S1C Fig.). Interestingly, although shRNAs against SR-A1 and SR-B1 did not affect HAdV-C5 gene transduction, these shRNAs reduced the IFNα/β response elicited by HAdV-C5, whereas knockdown of SR-B2 had essentially no effect (Fig 1G).

### Mouse SR-A6 facilitates binding of HAdV-C5 to cells

**Fig 2:**
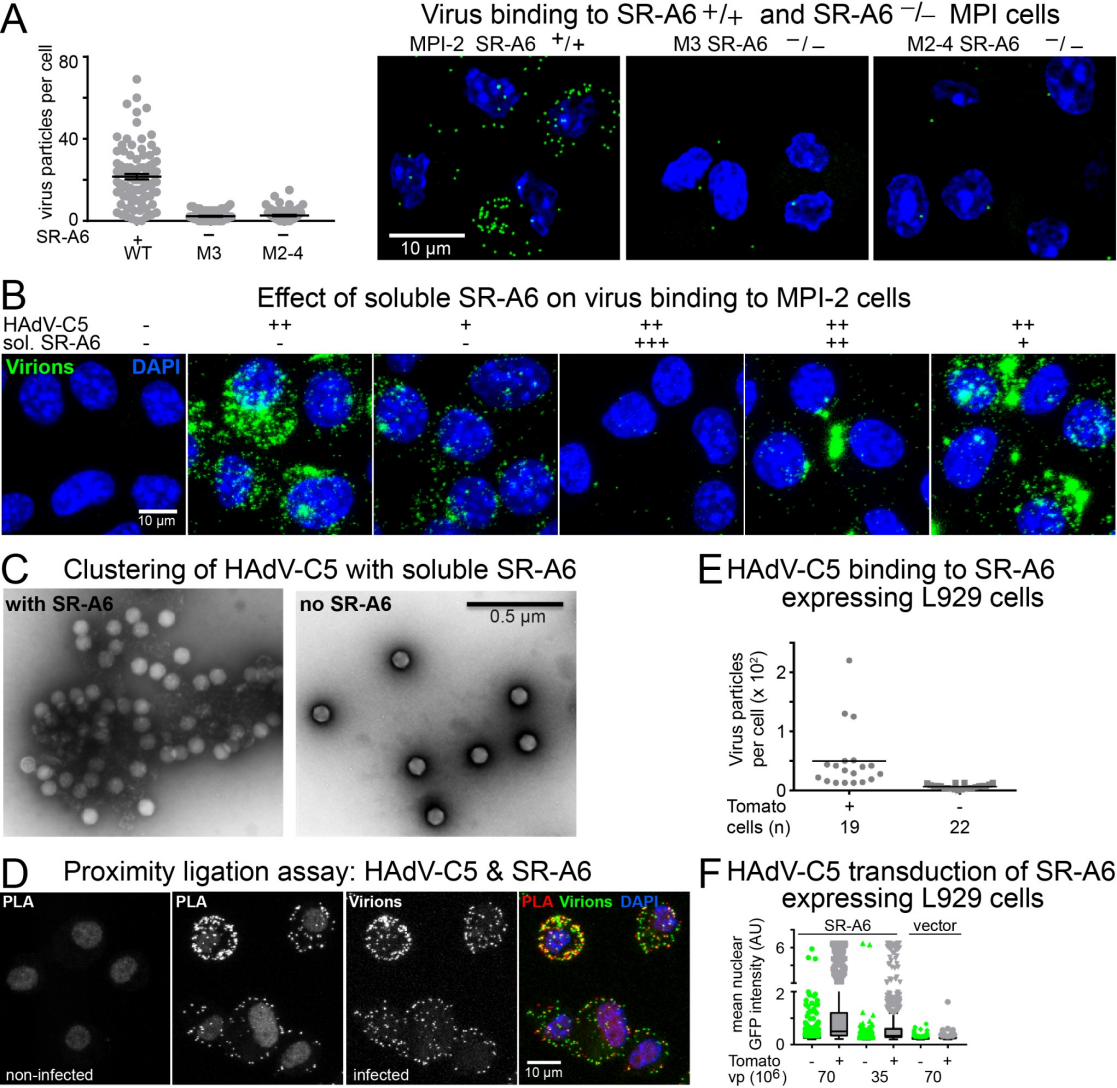
SR-A6 facilitates virus binding to MPI-2 cells. A) Binding of Alexa-Fluor488-labeled HAdV-C5 to parental MPI-2 cells or the mSR-A6^-/-^ M3 and M2-4 cell lines. Virus was added to cells at +4°C for 60 min (moi ~ 2540 virus particles per cell) and cells were shifted to 37°C for 5min before analysis. The plot shows number of bound virus particles per cell, one dot representing one cell. Error bars represent the means ± SEMs. The difference between SR-A6-positive and SR-A6 knockout cells was statistically significant (P<0.0001, Kolmogorov-Smirnov test). The right-hand panel shows representative images from the three cell lines analyzed. Images are maximum projections of confocal stacks. Virus particles are shown in green and nuclei (DAPI) in blue. Scale bar = 10 μm. B) Effect of preincubation of HAdV-C5 with three different amounts of soluble extracellular domain of mouse SR-A6. Input virus ~44600 virus particles per cell with the soluble SR-A6. The soluble form of SR-A6 in a concentration-dependent manner either suppressed virus binding to cells or caused virus to bind to cells in clustered forms. Scale bar = 10 μm. C) Representative negative stain EM images of HAdV-C5 incubated in the presence or absence of soluble, partially trimeric SR-A6. Scale bar = 0.5 μm. D) Proximity ligation assay indicates co-localization of virus and SR-A6 at the cell surface. Alexa-Fluor488-conjugated HAdV-C5 was added to cells at 4°C for 60 min (moi ~1370 virus particles per cell), and proximity ligation assay was performed with anti-Alexa-Fluor488 antibodies and anti-SR-A6 ED31 antibody. Images shown represent maximum projections of confocal stacks. PLA indicates signal from the proximity ligation assay, virus panel shows the Alexa-Fluor488-labeled virus particles and the overlay panel demonstrates the overlap of PLA and virus signals. Nuclei (DAPI stain) are in blue. Scale bar = 10 μm. E) Exogenous expression of murine SR-A6 in L-929 cells (low level of CAR expression) promotes binding of HAdV-C5 to the cells. SR-A6 was expressed in the cells from a bi-cistronic mRNA which also directed the synthesis of Tomato from an internal translation initiation site. Alexa-Fluor488- labeled HAdV-C5 particles were bound to transfected L-929 cells at 4°C. Fixed samples were imaged by confocal microscopy. Virus particles associated with Tomato-positive and–negative cells were scored from maximum projections of confocal stacks. The plot shows number of virus particles per cell, one dot representing one cell. Horizontal bars represent mean values. Number of cells analyzed is indicated. The difference between Tomato-positive and -negative cells was statistically significant (P<0.0001, Kolmogorov-Smirnov test). F) Exogenous expression of murine SR-A6 in L-929 cells promotes HAdV-C5-mediated gene transduction. Transfected L-929 cells were infected with two different amounts of HAdV-C5_dE1_GFP, and nuclear GFP signals were scored at 24h post infection by microscopy. The plot shows mean nuclear GFP intensities for Tomato-positive and–negative cells as Tukey box plots. More than 700 cells were analyzed for each sample.

We next compared binding of Alexa-Fluor488-tagged HAdV-C5 to SR-A6^+/+^ MPI-2 cells and the SR-A6^-/-^ M3 and M2-4 cells. Equivalent amounts of the fluorophore-tagged virions were added to the SR-A6^+/+^ and SR-A6^-/-^ cell populations at 4°C for 1h and after removal of unbound virus, cells were switched to 37°C for 5 min before fixation. The samples were imaged by confocal microscopy, and maximum projections of confocal stacks were used to score virus particles associated with cells. Whereas the SR-A6-positive MPI-2 cells contained many HAdV-C5 particles, only occasional virions were associated with M3 and M2-4 SR-A6 knock-out cells (Fig 2A).

The virion binding assay was repeated in MPI-2 cells expressing shRNAs against various scavenger receptors. Only shRNA directed against SR-A6 significantly reduced HAdV-C5 binding, whereas cells expressing shRNAs against SR-A1, SR-B1, SR-B2, or the nontargeting shRNA SR-A6_C911 bound virus essentially as efficiently as parental MPI-2 cells (S2A and S2B Fig). Soluble SR-A6 was found to be partially trimeric, as reported previously [57]. Soluble SR-A6 either suppressed virion binding to cells or induced virions to bind to cells in a clustered form (Fig 2B). These effects were strongly dose-dependent. Virion clustering was also observed when purified soluble SR-A6 and HAdV-C5 were mixed and analyzed by EM (Fig 2C). In the absence of soluble SR-A6, virus particles were monodispersed, but in the presence of SR-A6 large clumps of particles were frequently observed. These clumps most likely formed through crosslinking of virus particles by the trimeric fraction of SR-A6. In contrast to HAdV-C5, HAdV-B3 bound to MPI-2 cells inefficiently (S2C Fig.), and remained monodispersed after incubation with the soluble SR-A6 (S2D Fig.). Taken together, these results suggest that SR-A6 is an attachment receptor for HAdV-C5 in MPI-2 cells.

As a further validation, we tested virus colocalization with SR-A6 at the MPI-2 cell surface by proximity ligation assay (PLA) using Alexa-Fluor488-labeled HAdV-C5, rabbit anti-Alexa-Fluor488 antibody and the rat anti-SR-A6 ED31 antibody. PLA generates a fluorescent signal if the two antigens are located within a 40 nm distance of each other. When HAdV-C5 was bound to MPI-2 cells at 4°C, a strong signal was obtained in the PLA assay, whereas cells without virions gave no signal (Fig 2D). Furthermore, when mouse L929 cells, which are low in CAR expression, were transfected with a plasmid that directed the synthesis of a bi-cistronic mRNA for mouse SR-A6 and, via an internal ribosome entry site, for the red fluorescent protein Tomato, the Tomato-positive cells bound Alexa-Fluor488-tagged HAdV-C5 particles more efficiently than Tomato-negative (and thus SR-A6 negative) cells with the mean cell-associated virus number being seven-fold higher in Tomato-positive than Tomato-negative cells (Fig 2E). In addition, the mean nuclear GFP signal was about four-fold higher in Tomato-positive cells than in Tomato-negative cells after HAdV-C5_dE1_GFP transduction, whereas the Tomato-positive and -negative cells had similar mean nuclear GFP intensities in the control empty vector transfection, and these intensities were significantly lower than the GFP signals in the Tomato-positive cells in the SR-A6 transfection (Fig 2F).

### Hexon is the viral ligand for SR-A6

**Fig 3:**
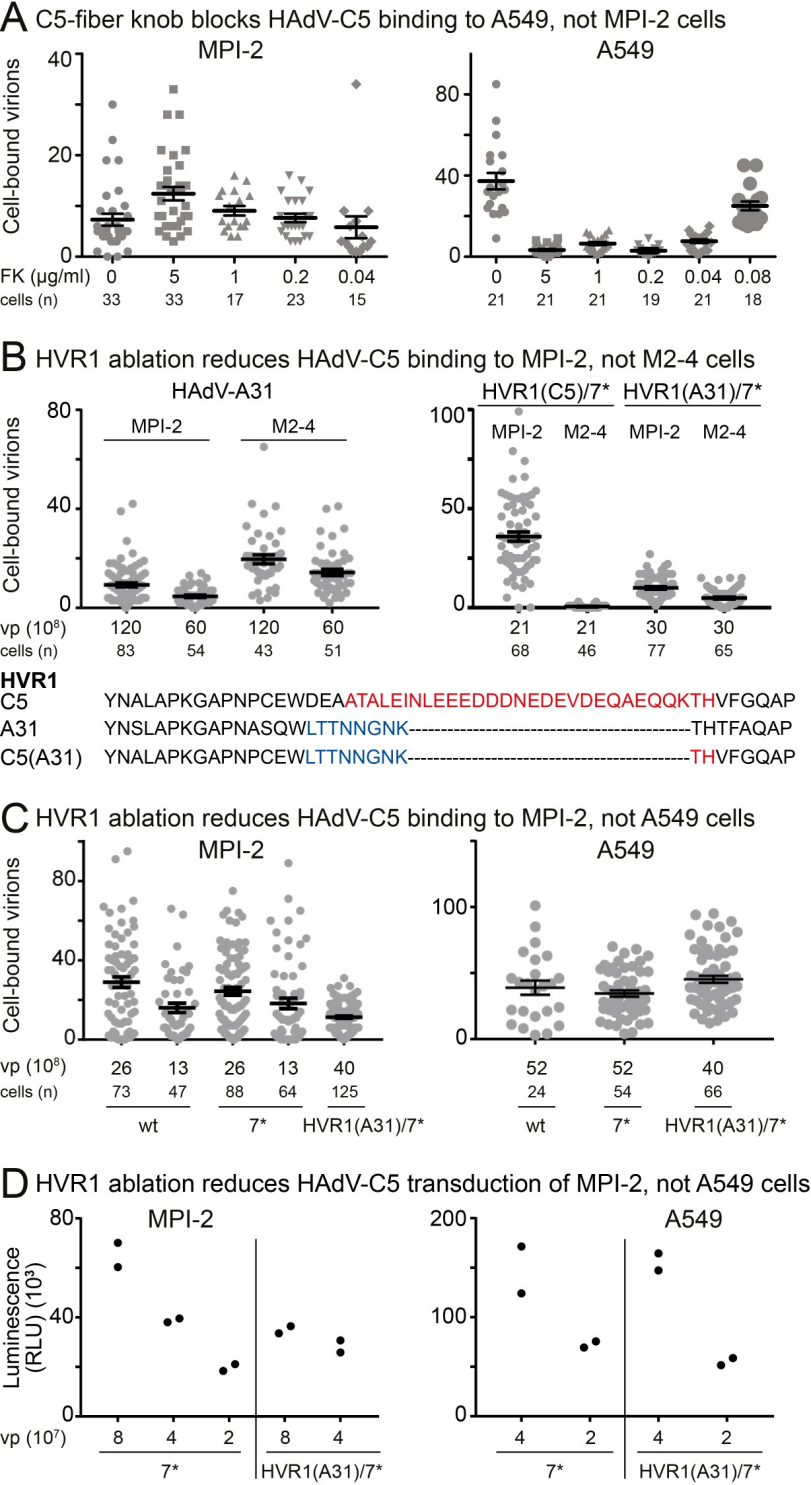
HVR1 on hexon controls binding of HAdV-C5 to MPI-2 cells. A) Pre-incubation of cells with soluble HAdV-C5 fiber knobs (FK) does not inhibit binding of HAdV-C5 to MPI-2 cells, whereas binding of the virus to the CAR-positive A549 cells is suppressed in a dose-dependent manner. Cells were first pre-incubated with the indicated amounts of FKs and Atto565-labeled HAdV-C5 was then added to the cells at 4°C for 60 min (moi ~ 20000 and 38000 virus particles/cell for MPI-2 and A549, respectively). After removal of unbound viruses and fixation, cells were imaged by confocal microscopy and cell-associated virus particles were scored from maximum projections of confocal stacks. The plot shows number of bound virus particles per cell, one dot representing one cell. Error bars represent the means ± SEMs. Number of cells analyzed is indicated. The difference between MPI-2 FK 0 and 5 μg/ml or 1 μg/ml samples was statistically significant (P = 0.002 and P = 0.032, respectively, Kolmogorov-Smirnov test). B) HAdV-A31 (with a truncated HVR1) binds better to SR-A6 negative M2-4 than MPI-2 cells, and HVR1 swap HAdV-C5_HVR1(A31)/HVR7* shows reduced binding to MPI-2 cells compared to HAdV-C5. The HVR1 swap was engineered into a virus backbone that carried single amino acid changes in hexon HVR7 (HVR7*), which prevent coagulation factor X binding to the virus. The experiment was carried out as described in (C). The difference between HAdV-C5_HVR1(A31)/HVR7* MPI-2 and M2-4 results, as well as that of HAdV-A31 results, is statistically significant (P<0.0001, Kolmogorov-Smirnov test). The HVR1 amino acid sequences of HAdV-C5 (highlighted in red), HAdV-A31 (blue) and HAdV-C5_HVR1(A31)/HVR7* (blue, red) are shown in single letter code. C) HVR1 ablation reduces HAdV-C5 binding to MPI-2 cells but not A549 cells. Indicated amounts (vp) of unlabeled wild type HAdV-C5, HAdV-C5_HVR7* and HAdV-C5_HVR1(A31)/HVR7* were added to ~ 1.6×10^5^ cells at 4°C for 60 min and after removal of unbound virus, cells were switched to 37°C for 10 min before fixation. Viruses were detected by immunostaining with an anti-hexon 9C12 antibody and secondary Alexa-Fluor488-conjugated antibody. Samples were imaged by confocal microscopy and cell-associated virus particles were scored from maximum projections of confocal stacks. The plot shows number of bound virus particles per cell, one dot representing one cell. Error bars represent the means ± SEMs. Number of cells analyzed is indicated. The difference between HVR1(A31)/HVR7* and the wild type or HVR7* viruses was statistically significant in MPI-2 cells (P = 0.0091 and P = 0.0006 for the vp 13×10^8^ samples, respectively, Kolmogorov-Smirnov test), whereas differences in A549 cells were not statistically significant. D) Swap of hexon HVR1 with that of HAdV-A31 reduces HAdV-C5 gene transduction in MPI-2 cells, but not in A549 cells. Cells (~4×10^4^) were infected with the indicated amounts of HAdV-C5_HVR7* and HAdV-C5_HVR1(A31)/HVR7* viruses and the activity of firefly luciferase expressed from the viral genomes was used to estimate the transduction efficiencies at 24 h pi. The plot shows results from two technical replicates.

To probe which virion-associated protein interacts with SR-A6, we first assayed whether binding of HAdV-C5 to the MPI-2 cells was mediated by the globular head domains (the knobs) of the vertex-associated fiber proteins, i.e. the same structures that mediate binding of the virion to the CAR receptor on non-immune cells. To this end, we incubated control untreated cells or cells preincubated with purified recombinant HAdV-C5 fiber knobs with Atto565-labeled HAdV-C5 at 4°C for 60 min, imaged fixed cells by confocal microscopy and used maximum projections of confocal stacks to determine the number of cell-associated virus particles. Whereas the fiber knobs efficiently suppressed virus binding to the CAR-positive A549 cells, no reduction of binding was evident in MPI-2 cells (Fig 3A). Instead, 5 μg/ml and 1 μg/ml FKs produced statistically significant increase of binding to MPI-2 cells (P = 0.002 and P = 0.032, respectively; Kolmogorov-Smirnov test). Of note, tagging of virus particles with different fluorophores somewhat affected HAdV-C5 binding to MPI-2 cells: whereas Alexa-Fluor488, which has an overall negative charge, increased binding in comparison to unlabeled viruses (e.g. Fig 2A and Fig 3B), Atto565 (no net charge) decreased binding (e.g. Fig 3A and 3B).

Ligand binding to SR-A6 and other SRs has been shown to be inhibited by polyanionic inhibitors such as poly G [58], thus suggesting that negatively charged residues on ligands are involved in the binding. Previously, the flexible HVR-containing loops on hexon have been implicated in binding of HAdV-C5 to the scavenger receptor SR-A1 [17]. The HVR1 of HAdV-C5 hexon is especially rich in negatively charged residues, as compared to HAdV-A31, for example (Fig 3B). Interestingly, binding of HAdV-A31 to MPI-2 cells was inefficient, whereas the SR-A6 negative M2-4 cells bound the virus more efficiently than MPI-2 cells (Fig 3B).

We tested the possible involvement of HVR1 in SR-A6 binding by engineering a recombinant HAdV-C5 in which the HVR1 was replaced by the HVR1 of HAdV-A31. This drastically shortened the HVR1 and removed the negatively charged residues (Fig 3B). The swap was done in HAdV-C5_HVR7* backbone that contained point mutations in HVR7, which ablate binding of virus to the coagulation factor X. The swap resulted in HAdV-C5_HVR1(A31)/HVR7*. As shown in Fig 3B and 3C, the point mutations in HVR7 did not affect binding of HAdV-C5 to MPI-2 cells, but the HVR1 swap significantly reduced virion binding, even when the HVR1-swapped virions were applied at higher concentration than wild type virions. In contrast, the HVR1 swapped virus bound to A549 cells as efficiently as wild type or the HVR7* control virus (Fig 3C). The concentrations of HAdV-C5_WT, HAdV-C5_HVR7* and HAdV-C5_HVR1(A31)/HVR7* were determined by absorbance measurements at 260 nm and verified by SDS-PAGE and silver staining (S3A Fig.). Representative images of fluorescent virions including HAdV-A31 particles on MPI-2, M2-4 (SR-A6 KO) and A549 cells are shown (S3B and S3C Fig.).

Similar to wild-type virus, efficient binding of HAdV-C5_HVR7* to MPI cells correlated with SR-A6 expression (Fig 3B). However, the difference in binding of HAdV-A31 or HAdV-C5_HVR1(A31)/HVR7* to the two cell types (MPI-2 and M2-4) was not as drastic as the reduction of binding of HAdV-C5_HVR1(A31)/HVR7* compared to HAdV-C5_HVR7* on MPI-2 cells. Note however that the HVR1-swapped virus still bound slightly more efficiently to MPI-2 cells than to the SR-A6 knock-out M2-4 cells (Fig 3B). The luciferase reporter gene in HAdV-C5_HVR7* and HAdV-C5_HVR1(A31)/HVR7* was used to probe the virus-mediated gene transduction. In agreement with the binding studies, the HVR1 swap reduced transduction by about two-fold in comparison to the HVR7* control, whereas the two viruses had similar transduction efficiencies in A549 cells (Fig 3D). Taken together, these results indicate that HVR1 of HAdV-C5 hexon contributes to interaction of the virus with SR-A6.

### Mouse SR-A6 promotes efficient HAdV-C5 entry into cells

**Fig 4:**
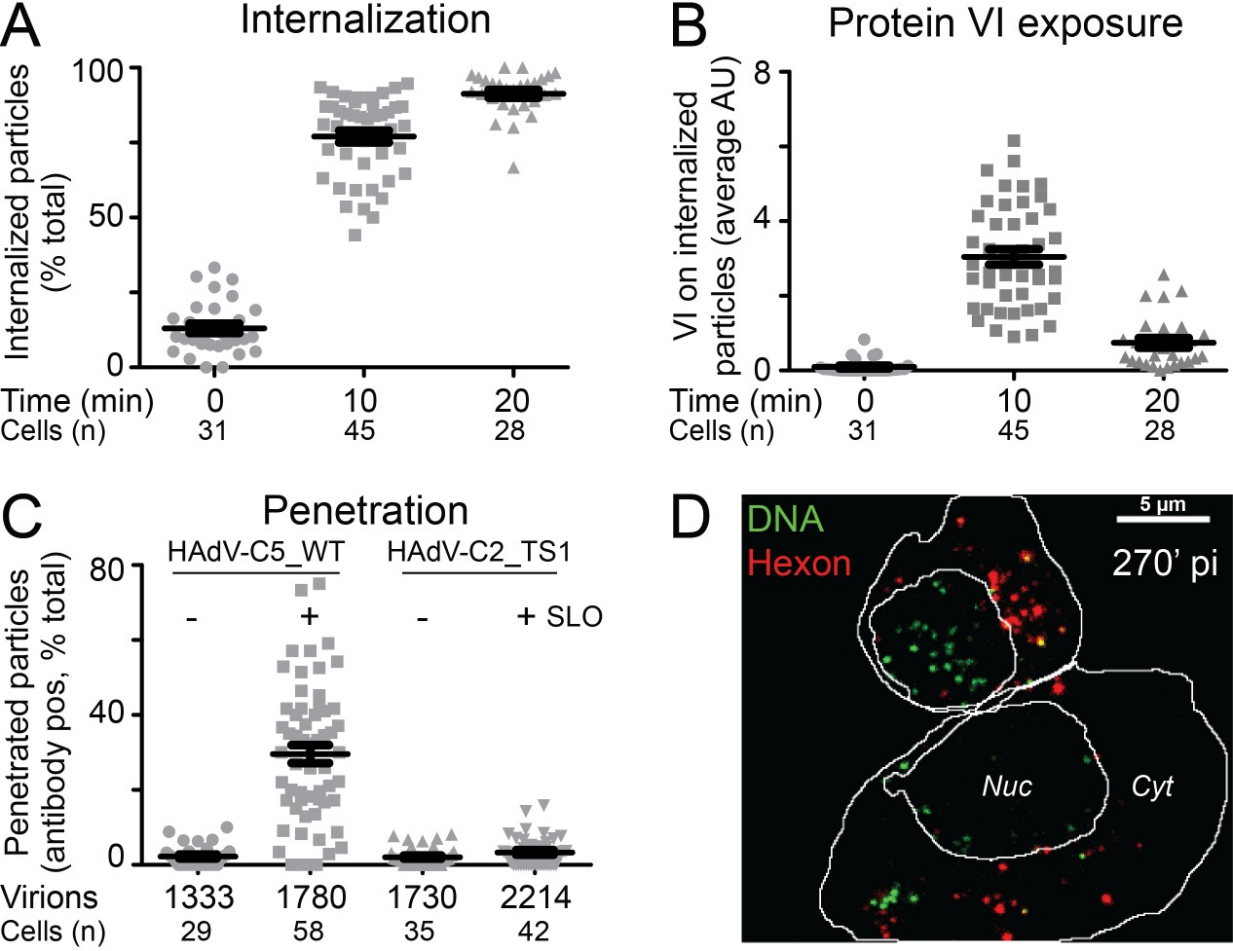
Characterization of HAdV-C5 entry into MPI-2 cells. A) Internalization of HAdV-C5 into MPI-2 cells and B) exposure of viral membrane lytic protein VI on internalized virus. Atto565-labeled HAdV-C5 were added to MPI-2 cells at 4°C (moi ~13600 virus particles per cell) for 60 min. Unbound particles were washed away, and cells were shifted to 37°C for the indicated times. Intact cells were then incubated with 9C12 anti-hexon antibodies at 4°C to tag surface-associated viruses, and after fixation, cells were permeabilized and stained for protein VI. Secondary Alexa Fluor680-conjugated anti-mouse and Alexa-Fluor488-conjugated anti-rabbit antibodies were used to detect 9C12 and anti-protein VI antibodies, respectively. Nuclei were stained with DAPI, and samples were imaged by confocal microscopy. Virus particles lacking the 9C12 signal were scored as internalized particles and (A) shows percentage of internalized virus particles per cell at the different time points. One dot represents one cell. (B) shows mean average protein VI signal on internalized particles. One dot represents one cell. Error bars represent the means ± SEMs. Number of cells analyzed is indicated. C) SR-A6-facilitated entry supports efficient penetration of HAdV-C5 into the cytoplasm. Alexa-Fluor488-conjugated HAdV-C5 were added to MPI-2 cells at 4°C for 60 min (moi ~7300 virus particles per cell). Unbound viruses were washed away and cells were shifted to 37°C for 45 min. Cell surface and cytoplasmic particles were tagged with anti-Alexa-Fluor488 antibodies after perforation of the plasma membrane with streptolysin O (SLO). The anti-Alexa-Fluor488 antibodies in turn were visualized by secondary Alexa-Fluor594 antibodies. Control cells were incubated with antibodies without SLO treatment to specifically mark virus particles at the plasma membrane. The plot shows percentage of virus particles positive for the anti-Alexa-Fluor488 antibodies, one dot representing one cell. The majority of antibody-positive particles in the SLO-treated HAdV-C wild type (wt) sample represent cytoplasmic virus, since the no-SLO control indicated only few particles at the cell surface. Virus particles in the endosomes are inaccessible to the antibodies, and the endosomes stayed intact in the assay, since parallel samples infected with the penetration deficient HAdV-C2-TS1 mutant virus displayed only low number of antibody-positive particles. Error bars represent the means ± SEMs. Numbers of cells and virus particles analyzed are indicated. D) Tracking of incoming virus genome. EdC-labeled HAdV-C5 particles (moi ~ 7300 virus particles per cell) were internalized into MPI-2 cells at 37°C for 30 min and, after removal of unbound virus, the samples were incubated for further 270 min before fixation. The virus capsids were visualized by anti-hexon 9C12 and Alexa-Fluor594-conjugated secondary antibodies, and click-reaction with Alexa-Fluor488-conjugated azide was carried out to mark the virus genomes. The image represents maximum projection of image stack from central parts of the cells. Nuclear area and cell outline are indicated. In the majority of cells, the virus genome was separated from the capsid at this time point and concentrated over the nuclear area, as exemplified by the upper cell in the image. A fraction of cells displayed significant amounts of capsid-free virus DNA also in the cytoplasm, as exemplified by the lower cell in the image. Scale bar = 5 μm.

To map the SR-A6-mediated entry pathway of HAdV-C5 in more detail, we analyzed the efficiency of SR-A6-mediated virus uptake and penetration into the cytoplasm. HAdV-C5 penetration into the cytoplasm is critically dependent on structural changes in the virion which lead to externalization of the viral membrane lytic protein VI [31, 32, 59, 60]. To monitor HAdV-C5 uptake and protein VI exposure in MPI-2 cells, Atto565-labeled HAdV-C5 was first bound to cells at 4°C to synchronize virus entry, and cells were then switched to 37°C for 0 min, 10 min or 20 min to allow internalization. After internalization, cells were switched back to 4°C and intact cells were incubated with the mouse 9C12 anti-hexon antibody to tag surface-associated virus. Fixed cells were stained with rabbit anti-protein VI antibody, and the 9C12 and anti-protein VI antibodies were detected by Alexa-Fluor680-conjugated anti-mouse and Alexa-Fluor488-conjugated anti-rabbit antibodies, respectively. Samples were imaged by confocal microscopy and the number of cell surface-associated virus particles (virus with both Atto565 and Alexa-Fluor680 signals) and internalized virus particles (virus with only Atto565 signal) were determined from maximum projections of the confocal stacks. As shown in Fig 4A, after cold binding and 0 min internalization, the majority of virions were identified as surface-associated particles. In contrast, after 10 min or 20 min incubation at 37°C, increasing numbers of virions were found to be internalized. Only occasional particles were positive for protein VI at 0 min, whereas the average protein VI intensity on internalized virus particles was significantly higher at the 10 min time point (Fig 4B and S4A Fig.), thus indicating that the particles had undergone structural changes during early steps of entry that enabled externalization of protein VI. The protein VI signal on internalized particles at 20 min post warming was reduced in comparison to the 10 min time point. Similar time-dependent reduction in particle-associated protein VI signal has been observed also during entry of HAdV-C5 into non-immune CAR-positive cells, an observation which reflects separation of protein VI from particles after penetration of the virus into the cytoplasm [31].

To assay the efficiency of virion escape into the MPI-2 cell cytoplasm more directly, we employed a streptolysin O (SLO)-based penetration assay [61]. Alexa-Fluor488-conjugated HAdV-C5 particles were bound to cells at 4°C, and subsequently internalized at 37°C for 45 min. After internalization, the plasma membrane was perforated by SLO to allow access of anti-Alexa-Fluor488 antibodies to the cytoplasm. These antibodies tag cell surface-associated and cytoplasmic virus particles, but particles in endosomes are inaccessible to the antibodies. The number of virus particles at the cell surface was estimated from parallel samples stained with anti-Alexa-Fluor488 antibodies without SLO permeabilization. After fixation, viruses tagged with the anti-Alexa-Fluor488 antibodies were scored by staining with secondary Alexa-Fluor594-conjugated antibodies. Samples were imaged by confocal microscopy and the number of virus particles positive or negative for the anti-Alexa-Fluor488 antibodies were scored from maximum projections of the confocal stacks. As shown in Fig 4C, approximately 30% of viruses were antibody-positive after 45 min of internalization, but the number of antibody-positive virus particles was variable between individual cells, as previously described for virus penetration in HeLa-Ohio and A549 cells [61]. The majority of the antibody-positive virions represented cytoplasmic particles, since staining of intact cells without SLO permeabilization gave only a low number of antibody-positive particles. The endosomes remained intact in the assay, since cells infected with the penetration-deficient, endosomal resident HAdV-C2_TS1 mutant virus exhibited only a low number of antibody-positive particles. Taken together, these results indicate efficient uptake and relatively efficient penetration of HAdV-C5 into the MPI-2 cells.

Incoming cytosolic HAdV-C5 particles traffic to the nucleus, disassemble at the nuclear pore complex and import their genome into the nucleus for viral gene expression and replication [62]. We used deoxy-5-ethynylcytidine-labeled (EdC) HAdV-C5 and copper(I)-catalyzed azide-alkyne cyclo-addition (click) reactions to monitor nuclear targeting of viral genomes in MPI-2 cells [63]. EdC-labeled HAdV-C5 was incubated with MPI-2 cells at 37°C for 30 min, and after washing unbound virus away, the cells were further incubated at 37°C for 30 min or 270 min before fixation. The viral capsids were visualized by anti-hexon 9C12 primary antibody and Alexa-Fluor594-conjugated anti-mouse antibodies, and the viral DNA was coupled with Alexa-Fluor488-conjugated azide. Representative images from the 30 min and 270 min time points are shown in Fig 4D and S4B Fig. At the early time point, the viral DNA was still extensively capsid-associated, but was largely uncoated at the 270 min time point, as indicated by the separation of DNA and capsid signals. In the majority of cells the uncoated DNA was concentrated over the nuclear area (identified by DAPI staining), but, as exemplified by the lower cell in Fig 4D, occasional cells had significant amounts of capsid-free DNA in the cytoplasm, as previously described for HeLa cells [63].

### SR-A6 facilitates infection of MPI-2 cells by HAdV-B35 and HAdV-D26

**Fig 5:**
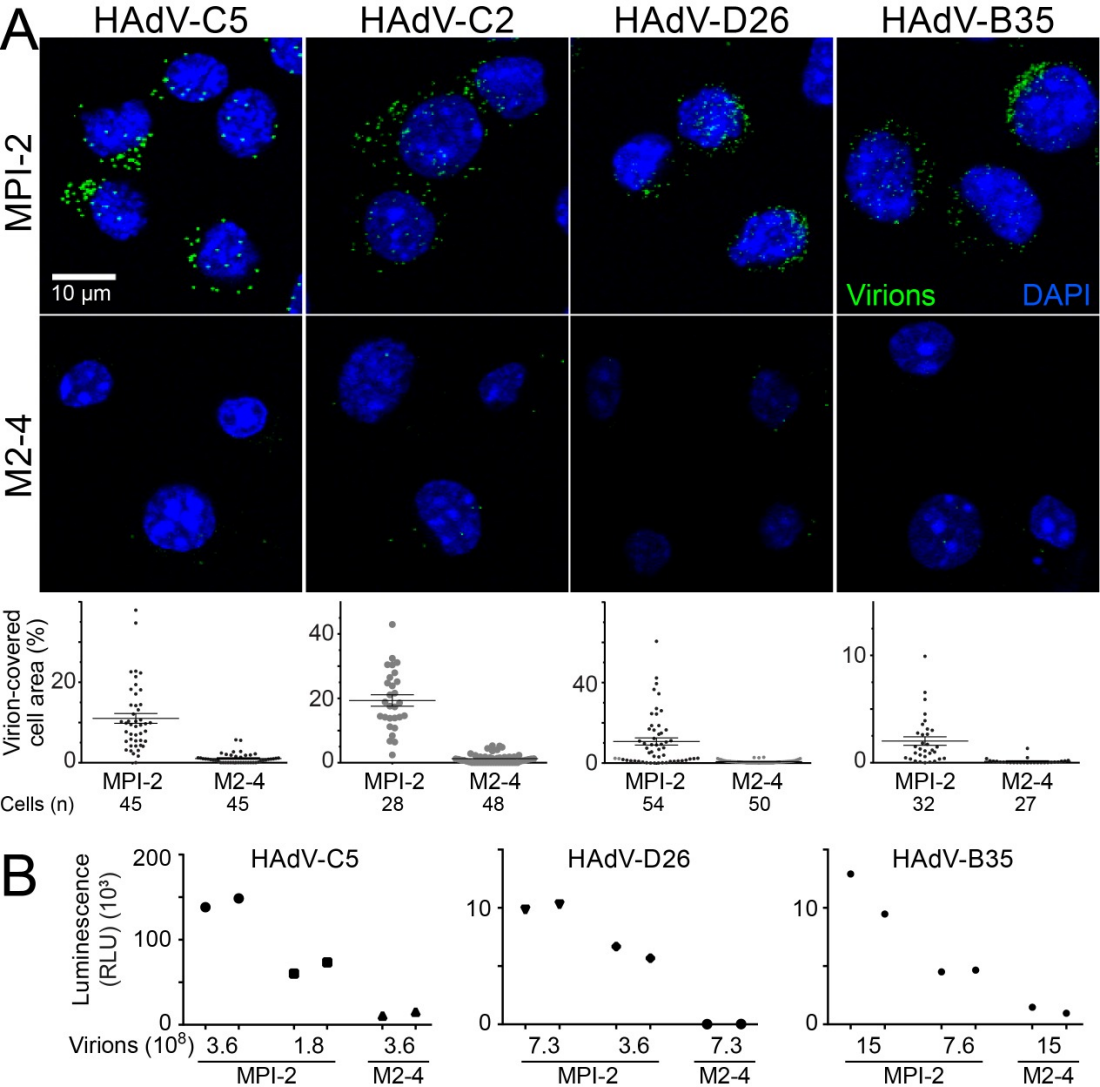
SR-A6 facilitates binding of HAdV-C2, HAdV-D26 and HAdV-B35 to MPI-2 cells. A) Alexa-Fluor488-labeled virus particles were added to wild type MP1-2 or to the SR-A6 knockout M2-4 cells as described in the legend to Fig 2A. Images shown are maximum projections of confocal stacks. Virus particles are pseudo-colored green and nuclei (DAPI) blue. Scale bar = 10 μm. The plots show quantification of virus binding efficiency, expressed as percentage of cell area covered by virus particles. Error bars represent the means ± SEMs, and number of cells analyzed is indicated. The difference in virus binding to MPI-2 and M2-4 cells was statistically significant for all viruses (P<0.0001, Kolmogorov-Smirnov test). B) SR-A6 facilitates HAdV-D26 and HAdV-B35 virus transduction. Wild type MPI-2 and the SR-A6 knockout M2-4 cells (~8×10^4^) were infected with indicated amounts of recombinant virus vectors carrying the Firefly luciferase gene, and the luciferase enzyme activity in cell extracts was used to estimate the virus transduction efficiencies at 20.5 h pi. The plot shows results from two technical replicates.

We were interested whether mouse SR-A6 is a receptor only for HAdV-C5, or whether the protein is a more general entry facilitator for different adenovirus serotypes. We compared binding of different adenoviruses to the MPI-2 and the SR-A6 M2-4 knockout cells. As shown in S2C Fig and Fig 3B, HAdV-B3 only inefficiently bound to MPI-2 cells and binding of HAdV-A31 did not correlate with SR-A6 expression. Efficient SR-A6-dependent binding was observed with Alexa-Fluor488-labeled subgroup B virus HAdV-B35 and subgroup D virus HAdV-D26, as well as with HAdV-C2 (Fig 5A). As judged from the mean number of virus particles per cell, HAdV-C5 binding to MPI-2 cells was approximately 3.6-fold more efficient than that of HAdV-B35 or HAdV-D26 (S5 Fig).

The luciferase reporter gene carried by HAdV-B35 or HAdV-D26 was used to assess transduction of wild type and SR-A6 knockout MPI cells by these viruses, in comparison to the HAdV-C5-luciferase virus. All three viruses transduced wild type MPI-2 cells more efficiently than the M2-4 SR-A6 knockout cells, although the relative transduction efficiencies of the three viruses differed (Fig 5B). Thus, SR-A6 facilitates not only HAdV-C5-mediated gene transduction, but also that of HAdV-B35 and HAdV-D26. However, since HAdV-B35 and HAdV-D26 cell binding was reduced only by about 3.6-fold in comparison to HAdV-C5, but the difference in gene transduction was about 20-fold or more, the entry steps downstream of cell attachment appear to occur with different efficiencies in C5 versus B35 and D26 HAdV infections.

### High surface expression of human SR-A6 facilitates HAdV-C5 infection

**Fig 6:**
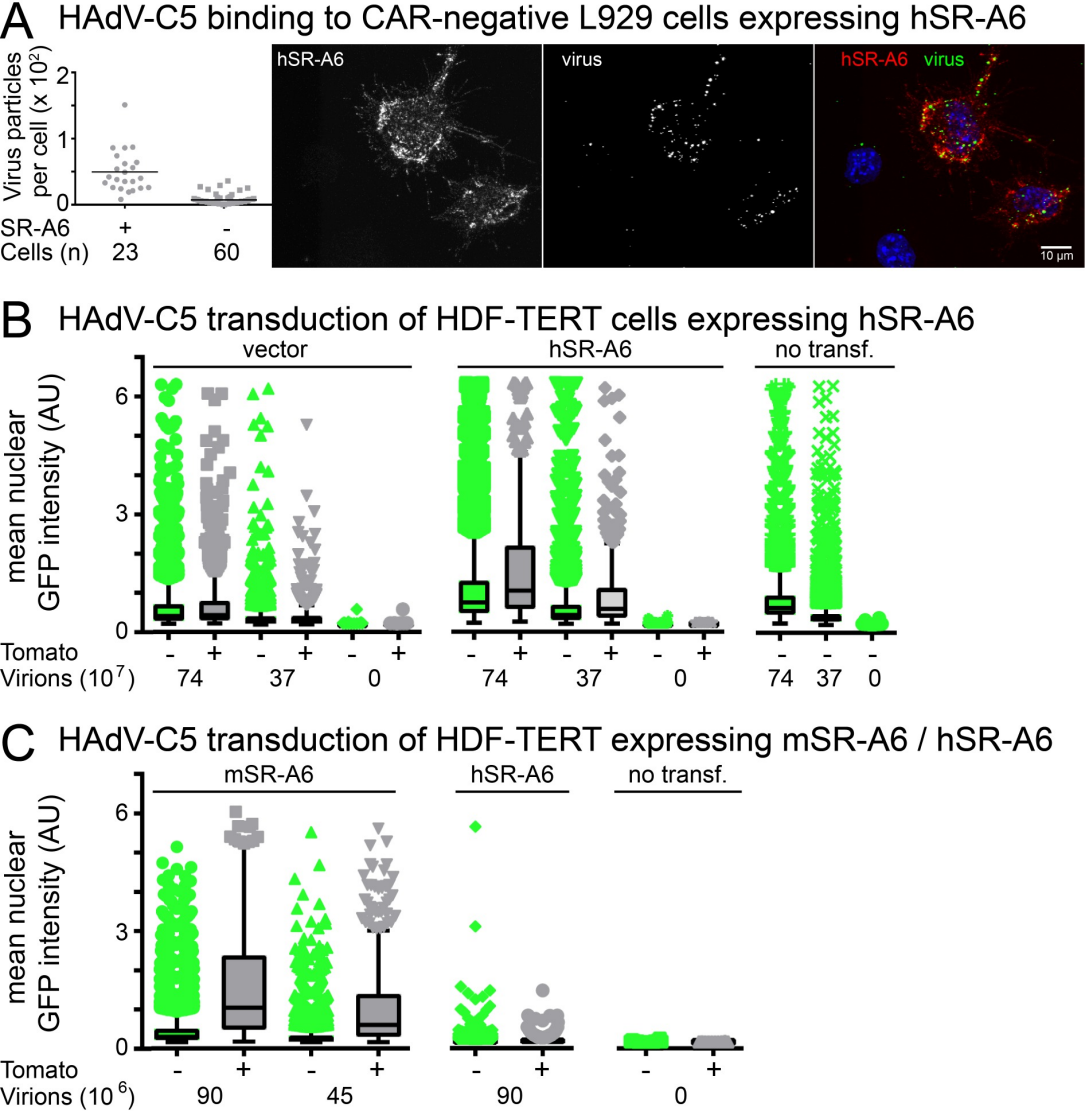
High surface expression of human SR-A6 facilitates HAdV-C5 infection. A) Exogenous expression of human SR-A6 in L-929 cells (low CAR expression) promotes binding of HAdV-C5 to the cells. L-929 cells were transfected with a plasmid directing the synthesis of human SR-A6 from the cytomegalovirus major immediate early promoter and transfected cells were identified by immunostaining with anti-human SR-A6 antibody PLK1. Alexa-Fluor488-labeled HAdV-C5 particles were added to transfected L-929 cells at 4°C for 60 min. Fixed samples were imaged by confocal microscopy and virus particles associated with PLK1-positive and PLK1-negative cells were scored from maximum projections of confocal stacks. The plot shows number of virus particles per cell, one dot representing one cell. Horizontal bars represent mean values. Number of cells analyzed is indicated. The difference between PLK1-positive and -negative cells was statistically highly significant (P<0.0001, Kolmogorov-Smirnov test). The right-hand panel shows a representative image as a maximum projection of confocal stacks. In the overlay panel virus particles are shown in green, SR-A6-positive cells in red and nuclei (DAPI) in blue. Scale bar = 10 μm. B) Exogenous expression of human SR-A6 in HDF-TERT cells boosts HAdV-C5-mediated gene transduction. SR-A6 was expressed in the cells from a plasmid that directed synthesis of the protein from a bi-cistronic SR-A6-IRES-Tomato mRNA. Transfected HDF-TERT cells were infected with two different amounts of HAdV-C5_dE1_GFP, and nuclear GFP signals were scored at 30 h post infection (pi) by microscopy. Non-transfected cells or cells transfected with an empty vector were used as controls. The mean nuclear GFP intensities of Tomato-positive and Tomato-negative cells are shown as Tukey box plots. The difference between Tomato-positive and Tomato-negative cells in the SR-A6 transfection was statistically highly significant with a P-value <0.0001 (Kolmogorov-Smirnov test), whereas the difference in empty vector transfections did not reach P<0.0001 significance levels. Over 300 Tomato-positive cells and more than 5000 Tomato-negative cells were scored for each sample. C) Mouse SR-A6 expression in HDF-TERT cells leads to higher HAdV-C5_dE1_GFP infection efficiency than human SR-A6 expression. Both mouse (mSR-A6) and human (hSR-A6) proteins were expressed from bi-cistronic SR-A6-IRES-Tomato mRNAs. The mean nuclear GFP intensities of Tomato-positive and Tomato-negative cells are shown as Tukey box plots. Over 500 Tomato-positive cells and more than 4500 Tomato-negative cells were scored for each sample.

To test whether human SR-A6 as well can act as a facilitator for HAdV-C5 infection, we first analyzed the ability of human SR-A6 to promote binding of Alexa-Fluor 488-conjugated HAdV-C5 to the low-CAR murine fibroblast-like L-929 cells. Plasmid expressing human SR-A6 from the cytomegalovirus major immediate early promoter was transfected into L-929 cells and the transfected cells were identified by immunostaining using the anti-human SR-A6 antibody PLK1. As shown in Fig 6A, cells expressing high levels of human SR-A6 at the surface bound the virus more efficiently than SR-A6-negative cells. However, we note that the increased virus binding was observed only in cells that stained strongly positive for SR-A6, whereas weak surface SR-A6 signal did not correlate with increased amounts of cell-associated virus.

As previously described [64], high SR-A6 expression induced dendritic surface projections and was very toxic to cells. Because of this high toxicity, we turned to a different cell line, the human diploid fibroblast-telomerase reverse transcriptase (HDF-TERT) immortalized cells, for testing the ability of human SR-A6 to boost HAdV-C5-mediated gene transduction. Compared to e.g. A549 cells, HDF-TERT cells bind HAdV-C5 inefficiently. Human SR-A6 was expressed in HDF-TERT cells from the same plasmid backbone as mouse SR-A6 in the L-929 cell experiments described above. The transfected Tomato-positive cells displayed variable levels of SR-A6 at the cell surface, which clearly correlated with the Tomato signal (S6 Fig.). The nuclear GFP signal from HAdV-C5_dE1_GFP transduction was used to assay infection efficiency in Tomato-positive/transfected and Tomato-negative/non-transfected cells. As shown in Fig 6B, Tomato-positive cells in human SR-A6 transfection exhibited enhanced nuclear GFP levels in comparison to Tomato-negative cells, or in comparison to Tomato-positive cells in the control empty vector transfection. However, this increase in nuclear GFP signal was rather modest, the mean nuclear GFP intensity in Tomato-positive cells was only ~ 1.6-fold higher than that in Tomato-negative cells, and the increase was observed only when relatively high levels of HAdV-C5_dE1_GFP were used for infection. This was in contrast to what was observed for exogenous expression of mouse SR-A6 in L-929 cells (Fig 2F). Therefore, we compared the abilities of mouse and human SR-A6 to boost HAdV-C5_dE1_GFP in HDF-TERT cells using the same plasmid backbone for expression of both proteins. As shown in Fig 6C, exogenous mouse SR-A6 expression facilitated HAdV-C5_dE1_GFP infection more efficiently than human SR-A6, as evidenced by ~ 8-fold higher mean nuclear GFP intensities in Tomato-positive cells in mouse SR-A6 transfection than in Tomato-positive cells in human SR-A6 transfection. Thus, although human SR-A6 can facilitate HAdV-C5 infection if expressed at high levels at the cell surface, human SR-A6 might be a less efficient receptor than the mouse SR-A6.

## Discussion

Macrophages defend an organism against insults, and are key for homeostasis, for example by engulfing apoptotic cells. Macrophages take up and present foreign antigens, kill pathogens, produce cytokines and chemokines, and coordinate tissue repair processes. Here we show that murine alveolar macrophage-like MPI-2 cells can be infected by human adenoviruses from several different species. We show that HAdV infection in MPI-2 cells requires the scavenger receptor SR-A6, and provide evidence that high expression levels of human SR-A6 can boost HAdV-C5 infection as well.

In the past, a number of different molecules have been implicated in HAdV attachment to mononuclear phagocytic cells, including MHC class 1, CD80/86, CD209 (DC-SIGN) and sialic acid [for a review, see 65]. *In vitro* and *in vivo* mouse studies have also implicated two other scavenger receptors, the SR-A1 (formerly named SR-A or SR-AI/SR-AII for its splice forms) and SR-F1/2 (SREC-1 or Scarf1) in the cell uptake of HAdV-C5 [16, 18], and exogenous expression of mouse SR-A1 (splice variant II) in CHO cells, which express low levels of CAR, has been shown to increase HAdV-C5 mediated gene transduction [16, 17]. The possibility that mouse SR-F1/2 functions as a HAdV-C5 receptor is based on the observation that hepatocyte transduction by intravenously applied HAdV-C5 vector was increased by prior treatment of the mice with anti-SR-F1/2 antibody Fab-fragments, the interpretation here being that the Fab fragments decreased virus entrapment by SR-F1/F2-positive Kupffer cells and liver sinusoidal endothelial cells, thus increasing hepatocyte transduction [18]. SR-F1/2 unlikely contributes to HAdV-C5 transduction of MPI-2 cells, since its mRNA was found to be expressed at higher levels in bone marrow derived macrophages (BMM) than MPI-2 cells, and BMM were largely resistant to HAdV-C5 [49, 53].

Here we show that the knockdown of SR-A1 did not reduce HAdV-C5 binding or virus-mediated gene transfer, whereas knockdown of SR-A6 drastically affected both binding and gene transduction in MPI-2 cells. This is in agreement with experiments using primary alveolar macrophages from SR-A1 knock-out mice which upon inoculation with Ad-GFP still expressed as high levels of GFP, as alveolar macrophages from wild type B6 animals, yet reduced IL-6 response was observed from the SR-A1 knock-out cells [53]. The apparent discrepancy between our study and the previous SR-A1 studies could stem from many factors, such as the cell surface expression levels, or expression of splice variants of SR-A1. Note that only the splice variant II of SR-A1 has been shown to facilitate HAdV-C5 entry. Intriguingly, although our results did not suggest a role for SR-A1 in HAdV-C5 binding or virus-mediated gene transfer in MPI-2 cells, IFNα/β response to virus was quenched upon SR-A1 (or SR-B1) knockdown, albeit not as drastically as upon SR-A6 knockdown. This suggests that SR other than SR-A6 could have roles in infection distinct from virion binding. Furthermore, as exemplified by HAdV-A31, surface receptors other than SR-A6 can mediate attachment of an adenovirus particle to macrophages, since binding of HAdV-A31 to MPI-2 cells did not correlate with SR-A6 expression. HAdV-A31 binding and transduction of macrophages were, however, clearly independent of SR-A6, indicated by the overall lower binding efficiency of HAdV-A31 on MPI-2 cells compared to HAdV-C5, even at 5-fold higher dose of HAdV-A31 than HAdV-C5.

HAdV-C5 infection of MPI-2 cells occurs through virion binding to the SR-A6 scavenger receptor, and leads to limited uncoating exposing the membrane lytic virion protein VI. Protein VI exposure enables virion penetration of an endosomal membrane and the transport of partly uncoated virions to the nuclear membrane, where the delivery of the viral DNA genome into the nucleus occurs. All these steps occur in the alveolar macrophage-like cells with high efficiency, albeit with significant cell-to-cell variation. The molecular basis of this variation is unknown at present. However, overall the endosomal escape was only slightly reduced compared to epithelial cells [61]. Epithelial cells engage the adenovirus particles by a sequential binding to CAR and integrin receptors, and exert mechanical cues to trigger the initial steps of uncoating [22, 24, 31, 32, 59–61, 66–68]. Whether integrins are also involved in entry of HAdV-C5 into MPI-2 cells is a subject for future studies.

The relatively efficient virus penetration into the cytoplasm of the MPI-2 cells not only enabled delivery of the viral genome into the nucleus for virus-mediated gene transduction, but also exposed the virus to cytoplasmic innate immune sensors, such as the cytoplasmic DNA sensor cyclic GMP-AMP synthase (cGAS) [53]. As previously described for HeLa cell infection [63], we observed that a fraction of HAdV-C5-infected MPI-2 cells displayed significant amounts of capsid-free virus genomes in the cytoplasm. This cytoplasmic DNA apparently plays a key role in innate immune sensing of incoming HAdV-C5 in MPI-2 cells, since HAdV-C5 entry into these cells triggers activation of the mitogen activated protein kinase p38, activation of the transcription factors IRF-3 and NF-κB, as well as secretion of interferon α/β, IL-6 and IL-1α, and these responses are blunted when cGAS is knocked-down in the cells [Fig 1G, and 53]. Collectively, our data support the ‘macrophage enigma’, where macrophages enable pathogens to replicate, and also serve as hubs to execute pathogen destruction and coordinate host defense [3].

SR-A6 is a trimeric disulfide-bonded, single-pass type II integral membrane protein, which belongs to the class A scavenger receptors [reviewed in 12, 58]. The extracellular domain of SR-A6 is composed of a collagenous triple helix structure and a carboxy-terminal type A scavenger receptor cysteine-rich domain. By using the mouse alveolar macrophage-like cell line MPI-2, we found several lines of evidence in support of SR-A6 being a receptor for HAdV-C5. First, knock-out of SR-A6 reduced binding of HAdV-C5 to MPI-2 cells, as well as virus-mediated gene transfer. Second, reduced HAdV-C5-mediated gene transduction was observed also when cells were pre-incubated with an anti-SR-A6 antibody. Third, a soluble form of mouse SR-A6 interfered with HAdV-C5 binding to MPI-2 cells and virus-mediated gene transfer, as well as induced virus clustering in an *in vitro* assay composed of purified soluble SR-A6 and HAdV-C5. Fourth, colocalization between surface-bound HAdV-C5 and SR-A6 on MPI-2 cells was observed in a proximity ligation assay, and, fifth, exogenous expression of mouse SR-A6 in the L-929 mouse cell line, which express low levels of CAR [69], increased binding of HAdV-C5 to these cells, as well as the virus-mediated gene transduction. Thus, mouse SR-A6 fulfills a set of stringent criteria for being a receptor of HAdV-C5. Its ability and to a lesser extent the ability of human SR-A6 to mediate binding and transduction with HAdV-C2, C5, B35 and D26 has implications for the transduction of human hematopoietic cells with B35, and human vaccinations with C5 and D26 [70–72]. Although the clustering of HAdV-C5 by soluble SR-A6 in the *in vitro* assay suggests that SR-A6 directly binds to this virus, the interaction is most likely of rather low affinity, similar to HAdV-B3 avidity binding to CD46 [29]. Weak binding of adenovirus to soluble SR-A6 is consistent with the notion that the trimeric form of SR-A6 is required for efficient ligand attachment [57].

Fiber knobs mediate interaction of HAdV-C5 with CAR, but binding of the virus to MPI-2 cells was not inhibited by soluble fiber knobs. In contrast, our results point towards hexon, and especially the HVR1 of hexon, being involved in attachment of HAdV-C5 to MPI-2 cells. When the long, negatively-charged HVR1 of HAdV-C5 was replaced by the short HVR1 of HAdV-A31, which lacks acidic residues, binding and gene transduction of the swap-virus in MPI-2 cells, but not in the CAR-positive A549 cells, was reduced in comparison to HVR1 wild type HAdV-C5. HVR1 is exposed on the surface near the rim of the cup formed in the hexon trimer (S7A Fig.), and is thus available for receptor interaction. In general, negatively-charged residues on SR-A6 and SR-A1 ligands have been implicated in mediating the binding to these SRs [17]. HAdV-A31 and HAdV-B3, which have short HVR1 and/or lack negatively-charged residue clusters in HVR1 (S7B Fig.) did not bind to MPI-2 cells efficiently or the binding did not correlate with SR-A6 expression, as shown for HAdV-A31. HAdV-B35 hexon has a cluster of acidic residues in HVR1, and binding of this virus to MPI cells did correlate with SR-A6 expression. However, although HAdV-D26 interaction with MPI cells also correlated with SR-A6 expression, a prominent acidic cluster in the D26 hexon HVR1 is missing (S7B Fig). Furthermore, although the hexon HVR1(A31) swap-HAdV-C5 exhibited reduced MPI cell binding, the mean cell-associated virus count in the binding assays was only reduced by about three-fold in comparison to HVR1 wild type virus. Thus, other surface-exposed hexon regions and/or other capsid-associated proteins may also contribute to the binding.

Like other scavenger receptors, SR-A6 binds to a number of ligands and/or exist in different complexes at the cell surface [11]. SR-A6 has been implicated to play a significant role in various host-pathogen settings in both mice and humans. For example, SR-A6-knockout mice show impaired clearance of bacteria from lungs and increased mortality upon *Streptococcus pneumoniae* infection [73]. In contrast, SR-A6 apparently suppresses protective early inflammatory responses to influenza virus and the SR-A6-knockout mice exhibit lower mortality upon influenza pneumonia than wild-type mice [74]. Furthermore, SR-A6 has been shown to modulate signaling from other pattern recognition receptors. For example, SR-A6 co-operates with Toll-like receptor 2 and CD14 to initiate innate immune responses to the *Mycobacterium tuberculosis* cell wall glycolipid trehalose dimycolate in mice [75], and SR-A6 knockout modulates proinflammatory responses to several different Toll-like receptor agonists in mouse splenic dendritic cells [76]. In addition, SR-A6 binds to the muramyl dipeptide (MDP), a cell wall component of both Gram-negative and Gram-positive bacteria, and knockout of SR-A6 reduces NOD2 (nucleotide-binding oligomerization domain 2)-mediated responses to the MDP, as well as NALP3 (NACHT domain-, leucine-rich repeat-, and pyrin-domain containing protein 3) inflammasome-dependent secretion of mature IL-1β upon challenge of mice with inactivated *Neisseria meningitides* [77]. SR-A6 is expressed on human lung macrophages and incubation of these cells with an anti-SR-A6 antibody reduced binding of latex beads, TiO_2_, heat-inactivated *E*. *coli* or *Staphylococcus aureus* to these cells, thus suggesting that SR-A6 has a major role in clearance of non-opsonized particles and bacteria by human lung macrophages [73]. Recent studies have also identified human SR-A6 as a receptor for herpes simplex type 1 and vaccinia virus [15, 78].

High expression levels of human SR-A6 upon transient transfection gave rise to HAdV-C5 binding and infection, albeit at low efficiency, whereas expression of mouse SR-A6 gave higher HAdV-C5 binding and infection levels. The human SR-A6 appears to be a less efficient receptor for HAdV-C5 than the mouse SR-A6. Possibly this is related to the differences in amino acids in the extracellular domains of human and murine SR-A6 [58]. Interestingly, polymorphism in the SR-A6 gene has been linked to increased susceptibility to pulmonary tuberculosis in humans [79, 80]. When transcriptional responses in primary monocytes from individuals of African or European origins were compared, SR-A6 was found to be among the genes displaying largest population differences upon stimulation with seasonal influenza A virus [81]. A function for mouse SR-A6 as a receptor for HAdV-C5 is consistent with previous observations that the mouse spleen marginal zone macrophages sequester HAdV-C5 after intravascular administration of the virion [44, 82, 83], and that SR-A6-positive lung macrophages trap HAdV-C5 in the respiratory tract [47, 48]. Overall, the study here identifies the scavenger receptor SR-A6 as an important transporter of HAdV into murine lung macrophages. This finding will enhance the interpretation of preclinical models in gene therapy and vector research, a field which is driven by the use of adenoviruses.

## Materials and methods

### Cells

The wild type (line 2) and SR-A6 deficient (lines M2-4 and M3) MPI (Max-Planck-Institute) alveolar macrophage-like cells were originally generated from fetal liver of wild type and SR-A6^-/-^ C57/BL6 mice [49, 53]. The cells were grown in RPMI 1640 supplemented with 10% heat-inactivated fetal calf serum (FCS; Gibco/Thermo Fisher Scientific) and 10 ng/mL murine recombinant GM-CSF (Miltenyi Biotec). Two different clones of human lung epithelial carcinoma A549 cells were used in the study: our old laboratory A549 clone and A549 from American Type Culture Collection (ATCC). Highly-polymorphic short tandem repeat loci profiling indicated that the two clones were ~95.1% similar. Human embryonic retinoblast (HER) 911 cells, which contain base pairs 79 to 5789 of the HAdV-C5 genome, were from American Type Culture Collection (ATCC) and maintained in DMEM supplemented with 7.5% FCS and 1% nonessential amino acids. Murine subcutaneous areolar and adipose tissue L-929 cells from ATCC, which express low levels of CAR, as well as the immortalized human diploid fibroblast HDF-TERT cells expressing the catalytic subunit of telomerase (kindly provided by Patrick Hearing and Kathleen Rundell) [84], were maintained in DMEM supplemented with 10% FCS and 1% nonessential amino acids. Mouse embryo fibroblasts (MEF) were obtained from Mx2-Luc IFN-β deficient (BKO), 13.5 days old Balb/c mouse embryos, as previously described for the MBa10 and MBa5 MEF [85]. For immortalization, primary cells were plated on 6-well cell culture dishes (10^5^ cells), and infected with two self-inactivating lentiviral vectors encoding for c-myc and E7 overnight in the presence of 8 μg/ml polybrene. The expression of the recombinant genes was driven by the SV40 promoter [86]. The following day infection medium was aspirated, and the cells were expanded with the above mentioned culture medium until they showed robust proliferation (doubling time ~1d) and the corresponding mock transduced primary cells stopped proliferating. The immortalized Mx2 luc BKO MEF, which express firefly luciferase under the interferon-inducible Mx2 promoter, were maintained in A549 base medium supplemented with 0.1 mM β-mercaptoethanol. HEK-293 cells producing recombinant, soluble mSR-A6 were a kind gift from Andrij Holian (University of Montana, USA) and maintained in A549 base medium with 250 μg/ml G418 and 1 μg/ml puromycin.

### Viruses

HAdV-C2 and HAdV-C5 were grown in A549 cells, whereas HAdV-A31 (kindly provided by Anja Ehrhardt, University of Witten/Herdecke, Germany) was grown in HeLa (ATCC) cells. The viruses were isolated, and labeled with Alexa-Fluor488 (Thermo Fisher Scientific) or Atto565 (Sigma-Aldrich) as described [87–89]. The replication-deficient HAdV-C5_dE1_GFP, an E1/E3 deletion mutant virus containing the enhanced green fluorescent protein (GFP) gene in the E1 region under the control of cytomegalovirus major immediate early promoter [90, 91] was grown in HER-911 cells. Notably, GFP localizes to the nucleus owing to a weak nuclear localization signal [92]. The replication-deficient HAdV-C5_HVR7* and HAdV-C5_HVR1(A31)/HVR7* with mutations in the hexon gene are derived from the AdEasy system (Agilent). The mutations were introduced in a 2-step process. First, the entire hexon gene was replaced by a kanamycin resistance gene flanked by two unique *Xba*I restriction sites using homologous recombination. In the second step, the kanamycin gene was removed by *Xba*I digestion. The mutated hexon gene was recombined with the linearized and purified pAdEasy fragment in BJ5183 [93], and selection for recircularization was performed using ampicillin. The negatively charged HVR1 of the hexon from HAdV-C5 was thus replaced by the short HVR1 from HAdV-A31 (DEAATALEINLEEEDDDNEDEVDEQAEQQ was changed to LTTNNGN). The modifications in HVR7 contained several point mutations which have been shown to ablate factor X binding (INTETL was changed to GNNSTY) [94]. The modified pAdEasy constructs were recombined with the plasmid pShuttle_CMV_luci_IRES_GFP (encoding firefly luciferase followed by a IRES and sfGFP). The viruses were grown in HEK-293 cells. All viruses were purified by two rounds of CsCl banding, dialyzed against 10 mM Tris pH 8.1, 150 mM NaCl and 1 mM MgCl_2_ and stored at -80°C in 10%(v/v) glycerol. Absorbance measurements at 260 nm or protein concentration were used for determination of number of virus particles per ml [95]. Alexa-Fluor488-labeled HAdV-D26 and HAdV-B35 vectors carrying the firefly luciferase gene were kindly provided by Jerome Custers and Dragomira Majhen (Janssen Pharmaceutical Companies of Johnson & Johnson, Netherlands).

### shRNA-mediated knockdown of mouse scavenger receptors

Plasmids pENTR/pTER+(431–1) and pQCXIN X2 DEST (w310-1) were a gift from Eric Campeau and obtained from Addgene (Addgene plasmids #17453 and #17399, respectively) [96]. Oligonucleotides used for cloning of the shRNA constructs targeting mouse scavenger receptors are listed in S1 Table. The oligonucleotide pairs were annealed and cloned via their BglII-HindIII-overhangs into the entry vector pENTR/pTER+(431–1), and recombined into the destination vector pCQXIN X2 DEST (w310-1) using Gateway cloning with LR clonase. Plasmids were verified by sequencing. VSV-G-pseudo-typed MLV retroviruses were generated according to standard procedure in HEK 293T cells. MPI-2 cells were infected with the retroviral vectors and after 48h treated with 0.5 mg/ml G418 to produce a polyclonal population of shRNA-expressing cells. A monoclonal cell population was generated for shSR-A6 cells by limiting dilution cloning.

### Viral transduction of MPI cells

Cells were seeded at a density of 30000 per well in a 96-well imaging plate and triplicate wells were infected the following day with three two-fold dilutions of HAdV-C5_dE1_GFP (300 ng, 150 ng or 75 ng virus per well) for 20h at 37°C in MPI-2 growth medium. The samples were fixed with 3% paraformaldehyde in phosphate-buffered saline (PBS) for 20 min at room temperature (RT), quenched for 10 min with 25 mM ammonium chloride in PBS and stained with a DAPI solution (1 μg/ml 4’,6-diamidino-2-phenylindole and 0.1% Triton X-100 in PBS). The plates were imaged on a Molecular Devices ImageXpress Micro XL high throughput microscope at a magnification of 10X and 3x3 sites per well. The infection was scored by measuring GFP-intensity over DAPI mask using a custom-programmed MatLab (The Mathworks) routine. The MatLab routines used in this study are available upon request.

For analyzing virus transduction efficiency by luciferase-assay, wild type MPI-2 or the SR-A6 knockout M2-4 cells were seeded at a density of 80000 cells per well in a 96-well plate and duplicate wells were infected the following day with HAdV-C5, HAdV-D26 or HAdV-B35 vectors carrying the firefly luciferase gene. At 20.5 h post infection (pi), cells were washed once with PBS, and cell extracts were prepared using 40 μl of Promega cell culture lysis reagent (# E153A) per well. Twenty-five μl of cell extracts were mixed with 35 μl of Promega Luciferase Assay Substrate (# E151A) and the luciferase activity in the cell extracts was recorded using Tecan Infinite 200 (Tecan, Switzerland).

### Virus binding assay in MPI cells

Cells were seeded at a density of 10^5^ or 2×10^5^ on coverslips in a 24-well plate format and grown for two days or one day, respectively. Alexa-Fluor488-labeled HAdVs (about 130 ng of HAdV-C5, or 1.8 × 10^9^ or 5.6 × 10^8^ virus particles in the case of HAdV-D26 and HAdV-B35, respectively) were added to cells at 4°C for 60 min in RPMI 1640 medium (without NaHCO_3_) supplemented with 0.2% bovine serum albumin (BSA; Sigma-Aldrich/Merck), 20 mM HEPES, and penicillin-streptomycin (RPMI-BSA medium). In the case of HAdV-A31 the virus was labeled with Atto565, whereas unlabeled virions were used in HAdV-C5_HVR1(A31)/HVR7* assays. Unbound particles were washed away and cells were switched to 37°C for 5 min. The cells were fixed and DAPI-stained as described above. Immunostaining with mouse anti-hexon 9C12 antibody [97], and secondary anti-mouse Alexa-Fluor488-conjugated antibodies were used to detect unlabeled viruses. Alexa-Fluor647 NHS Ester (A20006, Thermo Fisher Scientific: 0.5 μg/ml in PBS for 10 min) was used for staining of the cell area. Imaging was carried out with a Leica SP5 confocal laser scanning microscope using 63x magnification (oil immersion, numerical aperture 1.4) and zoom factor 2. Stacks were recorded at 0.5 μm intervals using 4× frame averaging, and a minimum of 50 cells per condition were imaged. A custom-programmed MatLab routine or a CellProfiler (http://cellprofiler.org) pipeline was used to determine the number of cell-associated virus particles from maximum projections of confocal stacks, or, if the number of cell-associated virus particles was too high for accurate single particle segmentation, the virus binding efficiency was estimated using a CellProfiler pipeline to determine the percentage of cell surface covered by virus particles. The resulting data were sorted using KNIME Analytics Platform (https://www.knime.org/knime-analytics-platform). GraphPad Prism (GraphPad Software, Inc. La Jolla) was used for producing scatter plots and statistical analyses. Representative images shown in figures are maximum projections of confocal stacks and images were processed with Fiji [98] applying the same changes in brightness and contrast to all image groups in the series.

### Blocking assays and soluble mouse SR-A6

For antibody-mediated blocking, cells were pre-incubated with rat-anti-mSR-A6 (clone ED31, Bio-Rad Laboratories, 1 mg/ml) at 1:25, 1:75 and 1:250 dilutions in 75 μl RPMI-BSA at 4°C for 30 min. Then, the above described virus transduction protocol was followed. Isotype-matched rat IgG1 negative control antibody was used as a control. The entire extracellular part of mouse SR-A6 (mSR-A6 soluble) was purified from transfected HEK-293 cells as described [57]. As judged from SDS-PAGE analyses, the protein was largely monomeric and contained a small amount of trimers (15–20%) in non-reducing SDS-PAGE analyses, as described earlier [57]. For soluble mSR-A6-mediated blocking, Alexa-Fluor488-labeled HAdV-C5 (about 800 ng) or HAdV-C5_dE1_GFP (about 300 ng) was pre-incubated with three different concentrations of the soluble mSR-A6 (about 125 ng, 250 ng or 500 ng) at room temperature for 15 min in 20 μl RPMI-BSA supplemented with 0.5 mM CaCl_2_ and 0.6 mM MgCl_2_, added to the cells in MPI growth medium and then the above described virus transduction and virus binding assay protocols using cells seeded on a 96-well plate were followed. For fiber knob (FK) experiments, MPI-2 and A549 cells grown on coverslips in 24-well plate (~1.6×10^5^ cells per well) were first preincubated for 30 min on ice with 8 ng/ml, 40 ng/ml, 200 ng/ml, 1 μg/ml or 5 μg/ml of soluble HAdV-C5 FKs [29] in RPMI-BSA medium, after which 1.6 μg of Atto565-conjugated HAdV-C5 was added, and incubation was continued for 45 min on ice. Unbound virus was washed away and cells were fixed and processed as described above for virus binding assay.

### Cell-based IFN-α/β measurement

MEF Mx2 luc BKO were seeded on 96-well plates the previous day at a density of 35000 cells per well. Medium was discarded and standards were applied in duplicates in 100 μl starting from 200 U/mL of recombinant mu-IFNβ (Sigma-Aldrich, #I9032) in seven two-fold dilutions. Fifty μl cell-free supernatants from infection experiments of MPI cells (see above) were combined with 50 μl fresh MEF-medium in duplicates and incubated for 20 h at 37°C. Then, medium was discarded and cells lysed with 40 μl of Cell Culture Lysis Reagent (Promega), incubated for 7 min on a rocker plate and 20 μl of each lysate was transferred to a LUMITRAC plate (Greiner). Forty μl Luciferase Assay Reagent (Promega, #E1500) was injected into each well on a TECAN plate reader with injection unit. After injection, the plate was shaken for two seconds and luminescence signal was integrated for 20 seconds. Values were background-subtracted, averaged, converted with an IFN-αβ calibration curve and plotted with GraphPad Prism.

### Flow cytometry

Cells were lifted by brief PBS/1.5 mM EDTA treatment, counted and divided into aliquots of 100000 cells each. Live cells were surface-stained with 1:10 diluted FITC-conjugated rat-anti-mSR-A6 (Bio-Rad Laboratories, #MCA1849FT, 0.1 mg/ml) on ice in PBS and co-incubated with propidium iodide according to the manufacturer’s recommendations (Molecular Probes). Data were collected on a FACS Canto flow cytometer (BD Biosciences) maintained by the Flow Cytometry Facility at the University of Zurich, and data were analyzed using FlowJo (TreeStar). Cells were gated on live singlets indicated by low propidium iodide incorporation and gating on FSC-H/FSC-A.

### qRT-PCR

Total cellular mRNA was isolated using a column-based Nucleospin II RNA Kit (Macherey-Nagel, #740995.50) as instructed by the manufacturer, followed by an extra DNaseI digest (Ambion, #AM1906). For qRT-PCR, a two-step protocol was employed. First, cDNA was synthesized from 150 ng total RNA in a volume of 20 μL using the Transcriptor First Strand cDNA Synthesis Kit (Roche, #05091284001) with an Oligo(dT)_18_ primer. Second, amplification was carried out in a total volume of 20 μl by using the MesaGreen SYBR Green Master Mix (Eurogentec, #RT-SY2x-03+WOU) in an ABIPrism 7900HT and pipetting by a TECAN Genesis robot. Oligonucleotides used are listed in S2 Table. Cycles consisted of an initial denaturation at 95°C for 5 min, forty cycles at 95°C for 15 sec and 60°C for 1 min, and a final dissociation step. All determinations were performed in technical triplicates. Controls lacking template and reverse transcriptase were run with every assay and had cycle thresholds (CT) which were significantly higher than experimental samples or undetermined. The relative abundance of each mRNA was calculated by the ΔΔCt method normalizing to three mouse housekeeping genes (GAPDH, TBP and EEF1A1).

### Proximity ligation assay

MPI-2 cells were seeded on coverslips at 200000 cells per well in a 24-well plate. Alexa-Fluor488-conjugated HAdV-C5 (70 ng) was added to cells in RPMI-BSA medium at +4°C for 60 min. Unbound viruses were removed by washing with cold RPMI-BSA medium, followed by fixation with 3% paraformaldehyde in PBS. Cells were then stained with rabbit anti-Alexa-Fluor488 antibody (A-11094, Thermo Fisher Scientific) and with rat anti-mSR-A6 ED-31 antibody (MBS215280, MyBioSource) for one hour at +4°C in a humidified chamber. Anti-mouse-MINUS (cross-reactive to rat antibodies) and anti-rabbit-PLUS PLA probes (conjugated with oligonucleotides) were added, and hybridization, ligation, amplification and detection steps were performed according to the manufacturer’s instructions (PLA Duolink, Olink) to generate an amplified fluorescent signal in areas where the antigens recognized by the two primary antibodies reside within less than 40 nm distance of each other. Fluorescent PLA signals were imaged using confocal fluorescence microscopy and the shown images represent maximum projections of confocal stacks.

### Expression of murine SR-A6 in L-929 cells

Murine SR-A6 was expressed from a cytomegalovirus major immediate early promoter as a bi-cistronic mRNA together with an internal ribosome entry site followed by tandem-dimer Tomato (the pCMVspit-vector construct was a kind gift from Peter Nielsen, Max-Planck-Institute of Immunobiology and Epigenetics, Freiburg Germany) [53]. The cDNA of murine SR-A6 contains both of the 5’ ATG codons [99]. The plasmid was transfected into L-929 cells by the Neon electroporation device (Thermo Fisher Scientific), using 5 μg of plasmid per 10^6^ cells and settings 1400 V, 30 ms, and 1 pulse. Empty vector was used as a control. After electroporation, cells were diluted into L-929 growth medium and plated on coverslips in 24-well plates for virus binding studies or on 96-well imaging plates for virus transduction studies. Twenty-four hours post transfection, virus binding was analyzed with Alexa-Fluor488-conjugated virus (~ 3 μg virus/well), and virus transduction was analyzed using HAdV-C5_dE1_GFP (17.5 or 8.8 ng virus/well) as described above, except that images from the virus transduction assay were analyzed by CellProfiler and the resulting data were sorted using the KNIME Analytics Platform. Since Tomato signals had somewhat different overall intensities in SR-A6 and empty vector-transfected cells, the threshold for Tomato-positive and Tomato-negative cells was adjusted for each sample so that about 10% of cells were scored as Tomato-positive, which roughly corresponded to the overall transfection efficiency. Mean nuclear GFP intensities were calculated for Tomato-positive and–negative cells and these intensities were used to score the infection. The CellProfiler and KNIME pipelines used will be made available upon request. GraphPad Prism was used for producing the scatter blots or Tukey box plots, as well as for statistical analyses.

### Expression of human SR-A6 in L-929 and HDF-TERT cells

Human SR-A6 cDNA was a kind gift from Dr. Peter Nielsen. It was expressed in L-929 cells from a modified pcDNA3.1-vector (ThermoFisher Scientific) in which the endogenous cytomegalovirus promoter was replaced by the cytomegalovirus immediate early promoter plus intron A (the plasmid was kindly provided by Jovan Pavlovic, University of Zurich). The cDNA of human SR-A6 starts from the second 5’ ATG codon, i.e. the expected main codon for the initiator methionine [99]. A cDNA that retained both of the 5’ ATG codons was also tested, but no improvement for HAdV-C5 binding to cells or virus-mediated gene transduction was observed with this variant. The plasmid was transfected into L-929 cells by Neon transfection as described above, except that 2.5 μg of plasmid was used per 10^6^ cells. Alexa-Fluor 488-conjugated HAdV-C5 (~ 5 μg virus/well) was used for analysis of virus binding to cells as described above. Transfected cells were identified by staining for surface SR-A6 using anti-human SR-A6 antibody PLK1 (HM2208, Hycult Biotech) and Alexa-Fluor 594-conjugated secondary anti-mouse antibodies as described [61]. The expression vector pCMVspit was used for expression of human SR-A6 in HDF-TERT cells. The vector was introduced into these cells by Neon transfection using 2 μg of plasmid per 5×10^5^cells and settings 1650 V, 10 ms and 3 pulses. Thirty-six hours post transfection cells were infected with HAdV-C5_dE1_GFP (190 or 95 ng virus/well) and virus infection was scored after 30 h as described above for mouse SR-A6-expressing L929 cells. In Fig 5B, the highest nuclear “Tomato” signal from non-transfected cells was used as a threshold for identification of Tomato-positive cells, and, accordingly, 15% and 6% of empty vector or SR-A6 transfected cells, respectively, were identified as Tomato-positive cells. In Fig 5C, the Tomato signals in human SR-A6 transfection were about two-fold higher than in the mouse SR-A6 transfection and the threshold for Tomato-positive and Tomato-negative cells was adjusted for each sample so that about 10% of cells were scored as Tomato-positive.

### Endocytosis and protein VI exposure assays

MPI-2 cells were seeded on cover slips at 200000 cells per well in a 24-well plate the previous day. HAdV-C5 (1.4 μg/well), labeled with Atto565 was added to cells in RPMI-BSA medium for 60 min on ice. Cells were then washed with cold RPMI-BSA and transferred to 37°C for 0, 10 and 20 min. Afterwards, intact cells were incubated with 9C12 anti-hexon antibody in RPMI-0.2% BSA at 0°C for 1h to tag surface virus as described [61]. The 9C12 antibody, developed by Laurence Fayadat and Wiebe Olijve, was obtained from Developmental Studies Hybridoma Bank developed under the auspices of the National Institute of Child Health and Human Development. Cells were then fixed with 3% paraformaldehyde in PBS and stained with affinity-purified anti-VI antibodies [31] and Alexa-Fluor680 anti-mouse and Alexa-Fluor488 anti-rabbit and DAPI as described in [61]. Imaging was performed with a Leica SP5 confocal microscope as described in [61]. Maximum projections of confocal stacks were analyzed by a custom-programmed MATLAB routine to score the number of cell surface-associated virus (virus with both Atto565 and Alexa-Fluor680 signals) and internalized virus (virus with only Atto565 signal) per cell, as well as protein VI signal on internalized virus particles. GraphPad Prism was used for producing scatter plots and statistical analyses. Representative images shown in figures are maximum projections of the confocal stacks, and the images were processed with Fiji applying the same changes in brightness and contrast to all image groups in the series.

### Streptolysin O assay to detect cytosolic virions

MPI-2 cells were seeded on coverslips at 80000 cells per well in 24-well plates and grown over two nights. Alexa-Fluor488-labeled HAdV-C5 virus (0.3 μg / well) was bound to cells in RPMI-BSA medium at +4°C for 60 min. Unbound virus was removed by washing with cold RPMI-BSA and cells were shifted to 37°C for 45 min to allow virus internalization. During the last 15 min of internalization, cells were treated with methyl-β-cyclodextrin/cholesterol mix (Sigma C4951: final concentration 480μM) to improve binding of streptolysin O (SLO) to cells. SLO permeabilization and scoring of cytoplasmic virus particles by rabbit anti-Alexa-Fluor488 antibody was done as previously described [61].

### Analysis of incoming virion genomes

EdC-labeled HAdV-C5 was produced as described in [63]. MPI-2 cells were seeded on coverslips at 80000 cells per well in 24-well plate and grown over two nights. Virus (0.3 μg / well) was bound to cells for 30 min at 37°C in DMEM supplemented with 0.2% BSA, unbound virus was washed away and incubation was continued for further 30 min or 270 min in MPI-2 growth medium at 37°C. Cells were then fixed with 3% paraformaldehyde in PBS, stained with the anti-hexon 9C12 antibody and Alexa-Fluor594-conjugated anti-mouse antibodies as described in [61], and subsequently the copper(I)-catalyzed azide alkyne cycloaddition (Click) reaction with Alexa-Fluor488-azide was carried out as described in [63]. Nuclei were stained with DAPI and samples were imaged by Leica SP8 upright confocal laser scanning microscope using a 63 × objective (oil immersion; numerical aperture 1.4) and zoom factor 4.09. Excitations were at 405 nm (DAPI), 488 nm (viral DNA) and 552 nm (virions). Stacks were recorded at 0.5 μm intervals using 4 × frame averaging for DNA and virus channels, and sequential acquisition for the individual channels. Representative images shown in figures are maximum projections of the whole stacks (30 min time point) or the middle thirteen sections of the cells (270 min time point), and the images were processed with Fiji applying the same changes in brightness and contrast to all image groups in the series.

### Electron microscopy

HAdV-C5 and HAdV-B3 virions (about 300 ng) were incubated with soluble murine SR-A6 (about 200 ng) in 20 μl of 20 mM Tris-HCl pH 7.4 buffer containing 150 mM NaCl, 1 mM MgCl_2_ and 0.9 mM CaCl_2_ for 20 min at room temperature, spotted on carbon-coated glow-discharged copper-palladium grids, and stained with 2% uranyl acetate for a few seconds [100]. Digital micrographs were collected with a Gatan Orius SC1000 CCD camera on a Philips CM100 electron microscope operating at 100 keV with a nominal magnification of 46,000×.

## Supporting information

S1 Fig Analyses of scavenger receptor expression in MPI-2 cells. A) Analyses of surface expression of SR-A6 in wild type MPI-2 cells and in SR-A6 knockout M3 and M2-4 cells by flow cytometry. FITC-conjugated rat anti-mouse SR-A6 ED31 antibody was used in the assay. Graphs show histograms of Alexa-Fluor488 channel in overlay and single panels, as well as mean and median read-outs. B) Determination of SR-A1, SR-A6, SR-B1 and SR-B2 transcript levels in MPI-2, M3 and M2-4 cells by qRT-PCR. The transcript levels are normalized to the wild type MPI-2 cells by three house-keeping genes. C) Secretion of IFN α/β from HAdV-C5-infected parental MPI-2 cells or MPI-2 cells expressing either an shRNA directed against SR-A6 or a control C911 non-targeting shRNA. Culture media from the infected cells were titrated on a reporter cell line expressing the Firefly luciferase gene under the IFN-inducible Mx2 promoter.
(PDF)

S2 Fig Virus binding to scavenger receptor knockdown MPI-2 cells. A) Binding of Alexa-Fluor488-labeled HAdV-C5 to MPI-2 cells expressing shRNAs against the scavenger receptors SR-A1, SR-A6, SR-B1 or SR-B2. Viruses were added to cells for 60 min at 4° (moi ~2655 virus particles per cell). Quantifications of bound virus particles per cell and representative images (maximum projections of confocal stacks) are shown. The difference between no shRNA and shSR-A6 cells was statistically significant (P<0.0001, Kolmogorov-Smirnov test), but the differences between no shRNA and shSR-A1, shSR-B1 or shSR-B2 cells were not significant. Viruses are pseudo-colored green and nuclei (DAPI stain) blue. Scale bar = 10 μm. B) Binding of Alexa-Fluor488-labeled HAdV-C5 to MPI-2 cells expressing the non-targeting C911 control shRNA against SR-A6. Viruses were added to cells for 60 min at 4°C (moi ~8855 particles per cell). Quantifications of bound virus particles per cell and representative images (maximum projections of confocal stacks) are shown for each cell line. Viruses are pseudo-colored green and nuclei (DAPI stain) blue. Scale bar = 10 μm. C) Comparison of binding of Alexa-Fluor488-labeled HAdV-B3 to MPI-2 and A549 cells. Viruses were added to cells for 60 min at 4°C (MPI-2 ~43400 virus particles and A549 ~4270 virus particles per cell) and the binding efficiencies were analyzed from fixed cells by confocal microscopy. The images shown represent maximum projections of confocal stacks. The amounts of input virus are indicated. In the overlay panel viruses are pseudo-colored green and nuclei (DAPI stain) blue. Scale bar = 10 μm. D) HAdV-B3 remains mono-dispersed after incubation with soluble mouse SR-A6. Representative negative stain EM images of HAdV-B3 incubated in the presence or absence of soluble SR-A6. Scale bar = 500 nm.
(PDF)

S3 Fig Effect of hexon HVR1 on binding of HAdV-C5 to MPI-2 cells. A) HAdV-C5_wild type (WT), HAdV-C5_HVR7* and HAdV-C5_HVR1(A31)/HVR7* virus preparations were analyzed by SDS-PAGE (8% gel) and silver staining to verify virus concentrations determined by absorbance measurements at 260 nm. Virus amounts loaded on the gel are indicated, as well as the position of viral proteins II (hexon), III (penton base), IV (fiber) and V. B) Representative images showing binding of HAdV-C5_wild type (WT), HAdV-C5_HVR7* and HAdV-C5_HVR1(A31)/HVR7* virions to MPI-2 and A549 cells. Input virus amounts in MPI-2 cells were 26×10^8^ virions for HAdV-C5_wild type and HAdV-C5_HVR7*, and 40×10^8^ virions for HAdV-C5_HVR1(A31)/HVR7*, whereas 52×10^8^ virions of HAdV-C5_wild type and HAdV-C5_HVR7* or 40×10^8^ virions of HAdV-C5_HVR1(A31)/HVR7* were added to A549 cells at 4°C for 60 min. The images show maximum projections of confocal stacks. Virions are shown in green and DAPI-stained nuclei in blue. Scale bar = 10 μm. C) Representative images showing the effect of hexon HVR1 on binding of HAdV-C5 and HAdV-A31 to MPI-2 and M2-4 (SR-A6 knockout) cells. Input virus amounts for HAdV-C5_HVR7* and HAdV-C5_HVR1(A31)/HVR7* were 21×10^8^ and 30×10^8^ virions, respectively, and 120×10^8^ virions for HAdV-A31. The images show maximum projections of confocal stacks. Virions are shown in green (HAdV-C5_HVR7* and HAdV-C5_HVR1(A31)/HVR7*) or red (HAdV-A31), and DAPI-stained nuclei in blue. The Atto565 labeling caused partial clustering of HAdV-A31. Scale bar = 10 μm.
(PDF)

S4 Fig HAdV-C5 entry into MPI-2 cells. A) Representative images showing protein VI externalization upon virus entry into MPI-2 cells. The DAPI-stained nuclei are shown in blue. Scale bar = 10 μm. B) Representative images for tracking of incoming virus DNA in HAdV-C5-infected MPI-2 cells. The image for the 30 min time point is a maximum projection of a confocal stack through the entire cell volume. Nuclear and cell outlines are indicated. Empty capsid (red) signals in the nuclear area represent capsid remnants below or above the nucleus, whereas the nucleus-associated uncoated DNA (green) can signify either DNA imported into the nucleus, DNA associated with the cytoplasmic side of the nuclear envelope or DNA above or below the nucleus. For the 270 min time point image, confocal slices below and above the nucleus were excluded from the maximum projection, and thus the nucleus-associated uncoated DNA is expected to largely represent DNA imported into the nucleus. Scale bar = 5 μm.
(PDF)

S5 Fig Comparison of HAdV-C5, HAdV-B35 and HAdV-D26 binding to MPI-2 cells. Alexa-Fluor488-labeled viruses (moi ~1000 virus particles per cell) were added to cells at 4°C for 60 min, and after removal of unbound virus, cells were incubated at 37°C for 10 min before fixation. Cells were imaged by confocal microscopy and cell-associated virus particles were scored from maximum projections of confocal stacks. The plot shows number of bound virus particles per cell, one dot representing one cell. Error bars represent the means ± SEMs. Number of cells analyzed is indicated.
(PDF)

S6 Fig Surface expression of human SR-A6 correlates with Tomato signal in transfected HDF-TERT cells. Representative images showing surface expression of human SR-A6 in HDF-TERT cells transfected with a plasmid that directed the synthesis of a bi-cistronic SR-A6-IRES-Tomato mRNA. Control cells were transfected with the empty plasmid backbone. Forty hours post transfection intact cells were incubated with the anti-human SR-A6 PLK1 antibody at 0°C, fixed, incubated with Alexa-Fluor488-conjugated secondary anti-mouse antibodies and DAPI-stained. Images are maximum projections of confocal stacks. Transfected cells displayed variable levels of SR-A6 at the cell surface, and by visual inspection, the intensity of surface SR-A6 signal correlated with the intensity of the Tomato signal. No PLK1 antibody signal was detected on non-transfected Tomato-negative cells or on Tomato-positive cells in the control transfection. Scale bar = 10 μm.
(PDF)

S7 Fig Location of HVR1 on hexon and comparison of HAdV HVR1 sequences of viruses used in the study. A) Ad5 hexon (PDB ID code 6B1T) is depicted in blue and ribbon corresponding to residues WDEAATALEINLEEEDDDNEDEVDEQAEQQKTHVFGQ, including the HVR1 as defined by [1, 2], is highlighted in red using the molecular visualization program UCSF Chimera. The stretch of amino acids which appears to influence SR-A6 binding is exposed on the surface near the rim of the cup formed in the hexon trimer. B) The HVR1 hexon loops of HAdV-C2 and HAdV-C5 were assigned according to [1], and are indicated in red and acidic residues in green. The sequence alignment was done using Clustal Omega at EMBL-EBI [3].
(PDF)

S1 Table Oligonucleotides used for cloning of constructs expressing shRNAs against mouse scavenger receptors. (PDF)

S2 Table Oligonucleotides used for qRT-PCR. (PDF)

S1 References (DOCX)
