# Supplementary material for: Lung macrophage scavenger receptor SR-A6 (MARCO) is an adenovirus type-specific virus entry receptor
Source: PLoS Pathog. 2018 Mar 9;14(3):e1006914. doi: 10.1371/journal.ppat.1006914 (PMC5862501; doi:10.1371/journal.ppat.1006914)

S1 Fig

A anti-SR-A6-FACS of SR-A6+/+ and -/- MPI cells

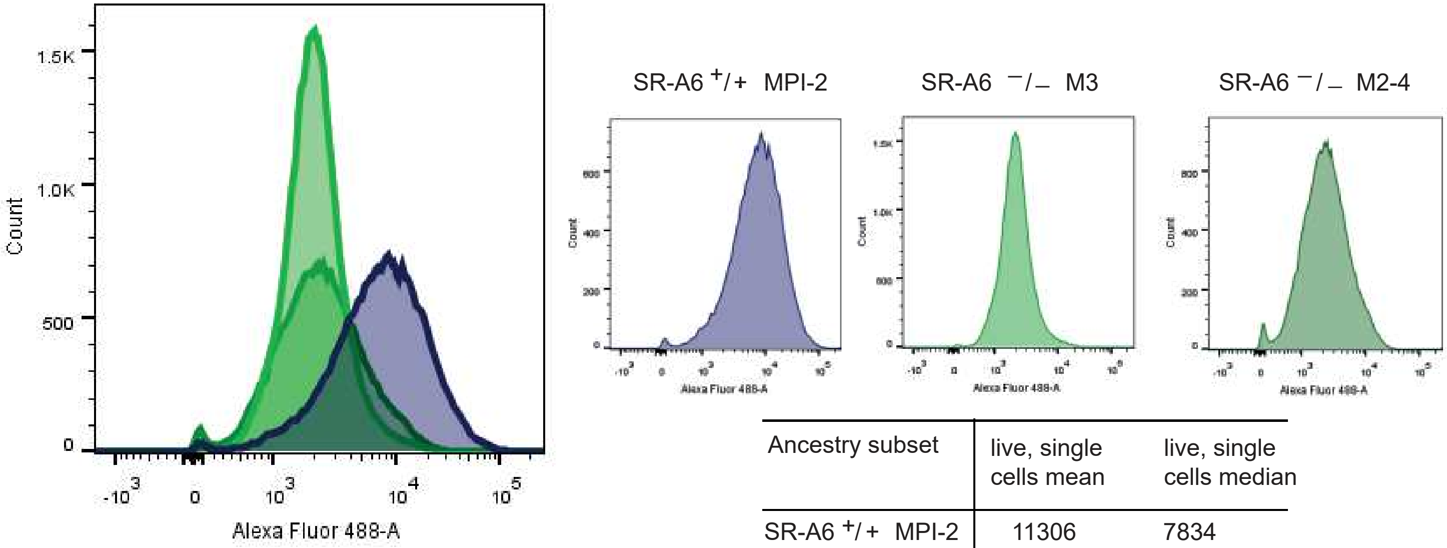

	Sample	Count
■	MPI-2	46919
■	SR-A6 -/- M3	57922
■	SR-A6 -/- M2-4	42277

Ancestry subset	live, single cells mean	live, single cells median
SR-A6 +/+ MPI-2	11306	7834
SR-A6 −/− M3	626	2152
SR-A6 −/− M2-4	3451	2361

B qRT-PCR of SR-A6+/+ and -/- MPI cells

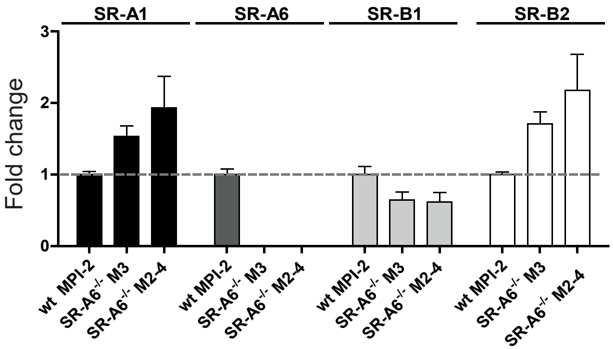

C Secretion of IFN α/β from control shRNA-transfected cells

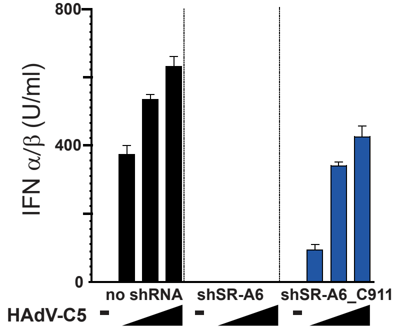

Supplement: S1 Fig — A) Analyses of surface expression of SR-A6 in wild type MPI-2 cells and in SR-A6 knockout M3 and M2-4 cells by flow cytometry. FITC-conjugated rat anti-mouse SR-A6 ED31 antibody was used in the assay. Graphs show histograms of Alexa-Fluor488 channel in overlay and single panels, as well as mean and median read-outs. B) Determination of SR-A1, SR-A6, SR-B1 and SR-B2 transcript levels in MPI-2, M3 and M2-4 cells by qRT-PCR. The transcript levels are normalized to the wild type MPI-2 cells by three house-keeping genes. C) Secretion of IFN α/β from HAdV-C5-infected parental MPI-2 cells or MPI-2 cells expressing either an shRNA directed against SR-A6 or a control C911 non-targeting shRNA. Culture media from the infected cells were titrated on a reporter cell line expressing the Firefly luciferase gene under the IFN-inducible Mx2 promoter. (PDF) [file ppat.1006914.s001.pdf]
