# Supplementary material for: Lung macrophage scavenger receptor SR-A6 (MARCO) is an adenovirus type-specific virus entry receptor
Source: PLoS Pathog. 2018 Mar 9;14(3):e1006914. doi: 10.1371/journal.ppat.1006914 (PMC5862501; doi:10.1371/journal.ppat.1006914)

S2 Fig

A HAdV-C5 binding to shSR-treated MPI-2 cells

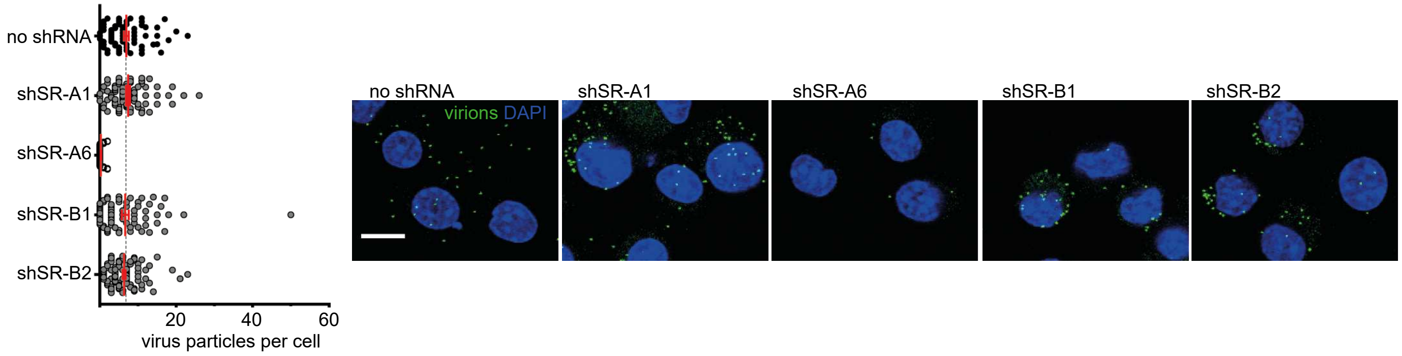

B Virion binding to shSR-A6_C911 MPI-2 cells

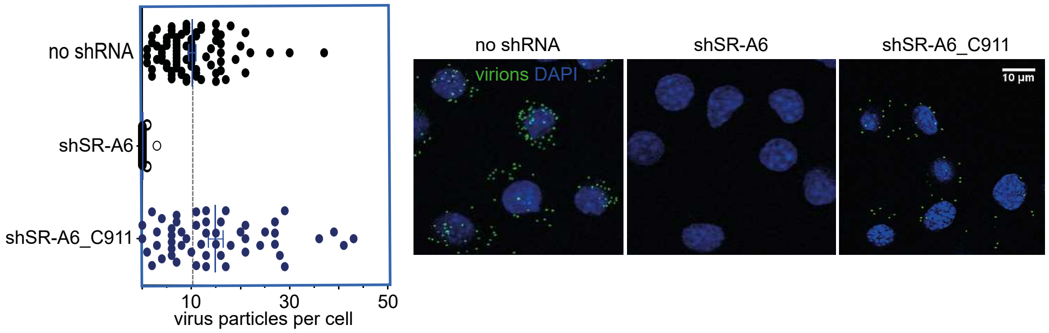

C HAdV-B3 binding to MPI-2 and A549 cells

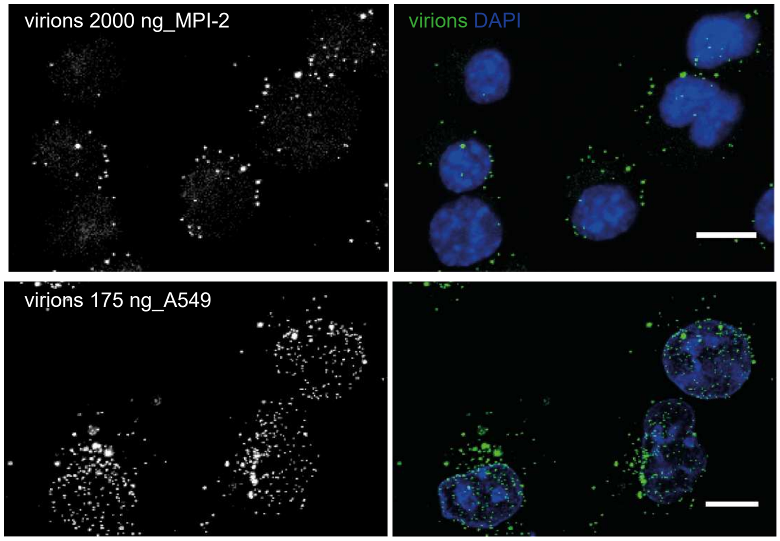

D HAdV-B3 incubated with soluble SR-A6

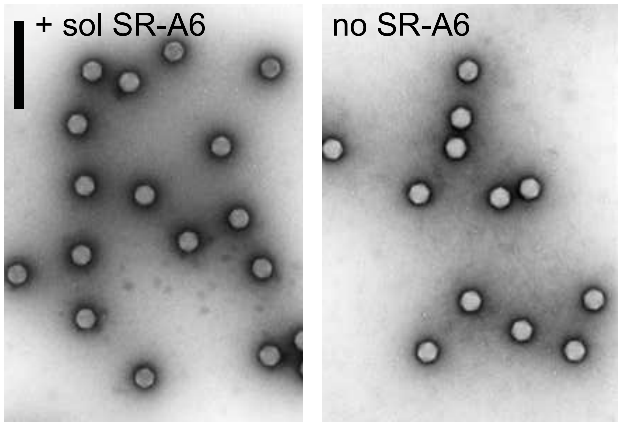

Supplement: S2 Fig — A) Binding of Alexa-Fluor488-labeled HAdV-C5 to MPI-2 cells expressing shRNAs against the scavenger receptors SR-A1, SR-A6, SR-B1 or SR-B2. Viruses were added to cells for 60 min at 4° (moi ~2655 virus particles per cell). Quantifications of bound virus particles per cell and representative images (maximum projections of confocal stacks) are shown. The difference between no shRNA and shSR-A6 cells was statistically significant (P<0.0001, Kolmogorov-Smirnov test), but the differences between no shRNA and shSR-A1, shSR-B1 or shSR-B2 cells were not significant. Viruses are pseudo-colored green and nuclei (DAPI stain) blue. Scale bar = 10 μm. B) Binding of Alexa-Fluor488-labeled HAdV-C5 to MPI-2 cells expressing the non-targeting C911 control shRNA against SR-A6. Viruses were added to cells for 60 min at 4°C (moi ~8855 particles per cell). Quantifications of bound virus particles per cell and representative images (maximum projections of confocal stacks) are shown for each cell line. Viruses are pseudo-colored green and nuclei (DAPI stain) blue. Scale bar = 10 μm. C) Comparison of binding of Alexa-Fluor488-labeled HAdV-B3 to MPI-2 and A549 cells. Viruses were added to cells for 60 min at 4°C (MPI-2 ~43400 virus particles and A549 ~4270 virus particles per cell) and the binding efficiencies were analyzed from fixed cells by confocal microscopy. The images shown represent maximum projections of confocal stacks. The amounts of input virus are indicated. In the overlay panel viruses are pseudo-colored green and nuclei (DAPI stain) blue. Scale bar = 10 μm. D) HAdV-B3 remains mono-dispersed after incubation with soluble mouse SR-A6. Representative negative stain EM images of HAdV-B3 incubated in the presence or absence of soluble SR-A6. Scale bar = 500 nm. (PDF) [file ppat.1006914.s002.pdf]
