# Supplementary material for: Lung macrophage scavenger receptor SR-A6 (MARCO) is an adenovirus type-specific virus entry receptor
Source: PLoS Pathog. 2018 Mar 9;14(3):e1006914. doi: 10.1371/journal.ppat.1006914 (PMC5862501; doi:10.1371/journal.ppat.1006914)
Supplement: S3 Fig — A) HAdV-C5_wild type (WT), HAdV-C5_HVR7* and HAdV-C5_HVR1(A31)/HVR7* virus preparations were analyzed by SDS-PAGE (8% gel) and silver staining to verify virus concentrations determined by absorbance measurements at 260 nm. Virus amounts loaded on the gel are indicated, as well as the position of viral proteins II (hexon), III (penton base), IV (fiber) and V. B) Representative images showing binding of HAdV-C5_wild type (WT), HAdV-C5_HVR7* and HAdV-C5_HVR1(A31)/HVR7* virions to MPI-2 and A549 cells. Input virus amounts in MPI-2 cells were 26×108 virions for HAdV-C5_wild type and HAdV-C5_HVR7*, and 40×108 virions for HAdV-C5_HVR1(A31)/HVR7*, whereas 52×108 virions of HAdV-C5_wild type and HAdV-C5_HVR7* or 40×108 virions of HAdV-C5_HVR1(A31)/HVR7* were added to A549 cells at 4°C for 60 min. The images show maximum projections of confocal stacks. Virions are shown in green and DAPI-stained nuclei in blue. Scale bar = 10 μm. C) Representative images showing the effect of hexon HVR1 on binding of HAdV-C5 and HAdV-A31 to MPI-2 and M2-4 (SR-A6 knockout) cells. Input virus amounts for HAdV-C5_HVR7* and HAdV-C5_HVR1(A31)/HVR7* were 21×108 and 30×108 virions, respectively, and 120×108 virions for HAdV-A31. The images show maximum projections of confocal stacks. Virions are shown in green (HAdV-C5_HVR7* and HAdV-C5_HVR1(A31)/HVR7*) or red (HAdV-A31), and DAPI-stained nuclei in blue. The Atto565 labeling caused partial clustering of HAdV-A31. Scale bar = 10 μm. (PDF) [file ppat.1006914.s003.pdf]

S3 Fig

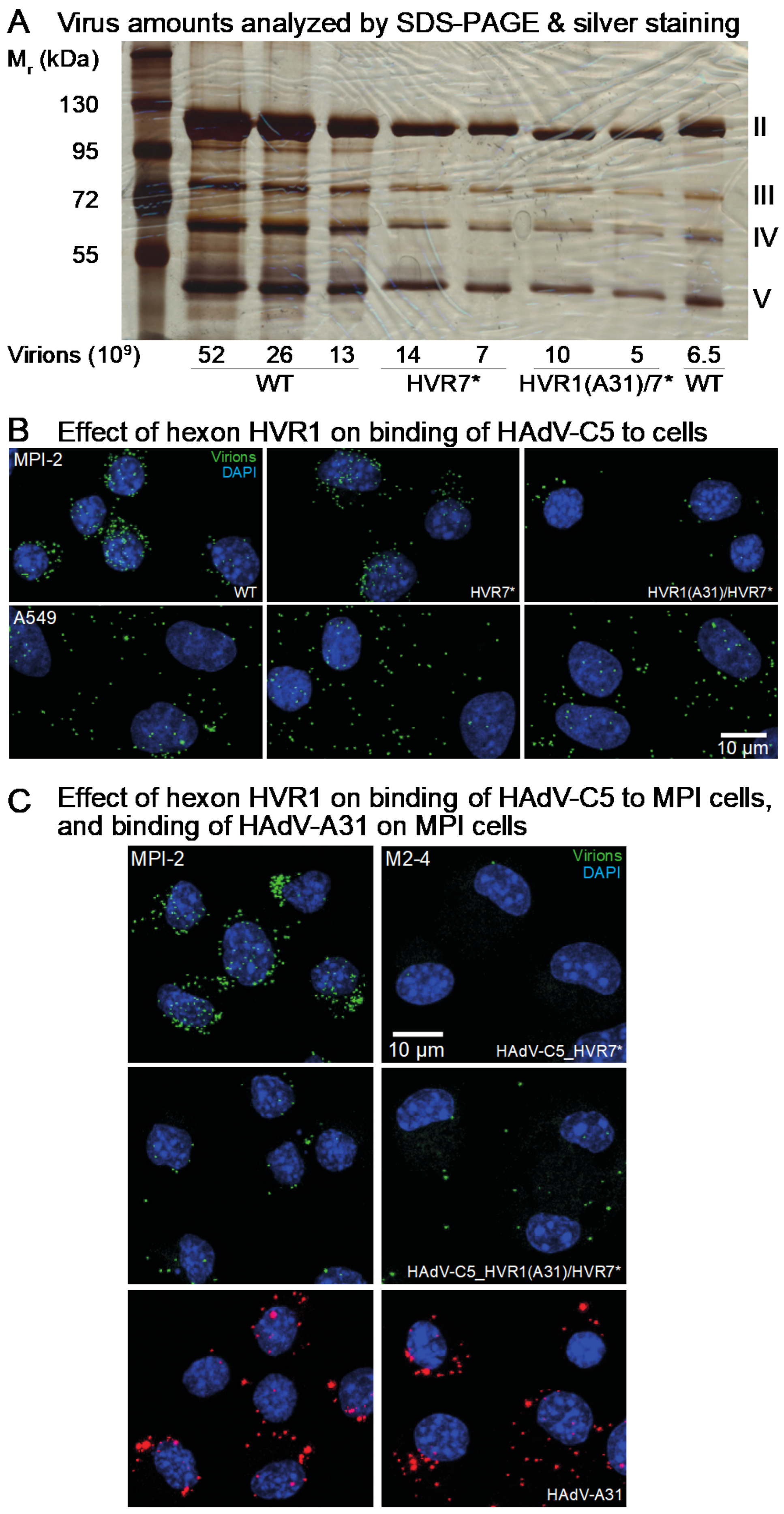
A
Virus amounts analyzed by SDS-PAGE & silver staining
Mr (kDa)
130
95
72
55
II
III
IV
V
Virions (10⁹)
52
26
13
14
7
10
5
6.5
WT
HVR7*
HVR1(A31)/7*
WT
B
Effect of hexon HVR1 on binding of HAdV-C5 to cells
MPI-2
Virions
DAPI
WT
HVR7*
HVR1(A31)/HVR7*
A549
10 µm
C
Effect of hexon HVR1 on binding of HAdV-C5 to MPI cells, and binding of HAdV-A31 on MPI cells
MPI-2
M2-4
Virions
DAPI
10 µm
HAdV-C5_HVR7*
HAdV-C5_HVR1(A31)/HVR7*
HAdV-A31
