# Supplementary figures and images for: Lung macrophage scavenger receptor SR-A6 (MARCO) is an adenovirus type-specific virus entry receptor
Source: PLoS Pathog. 2018 Mar 9;14(3):e1006914. doi: 10.1371/journal.ppat.1006914 (PMC5862501; doi:10.1371/journal.ppat.1006914)

## S4 Fig

### A Protein VI exposure

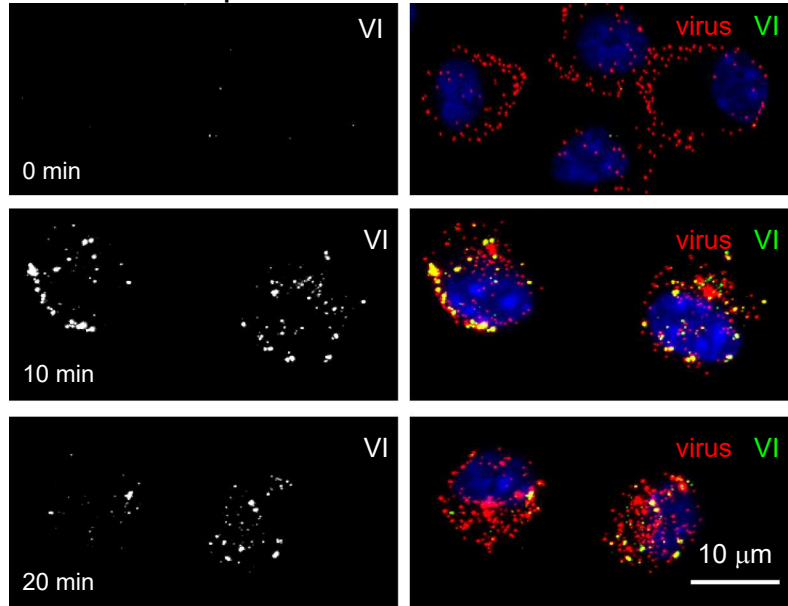

### B Incoming HAdV-C5 DNA detected by Click-reaction

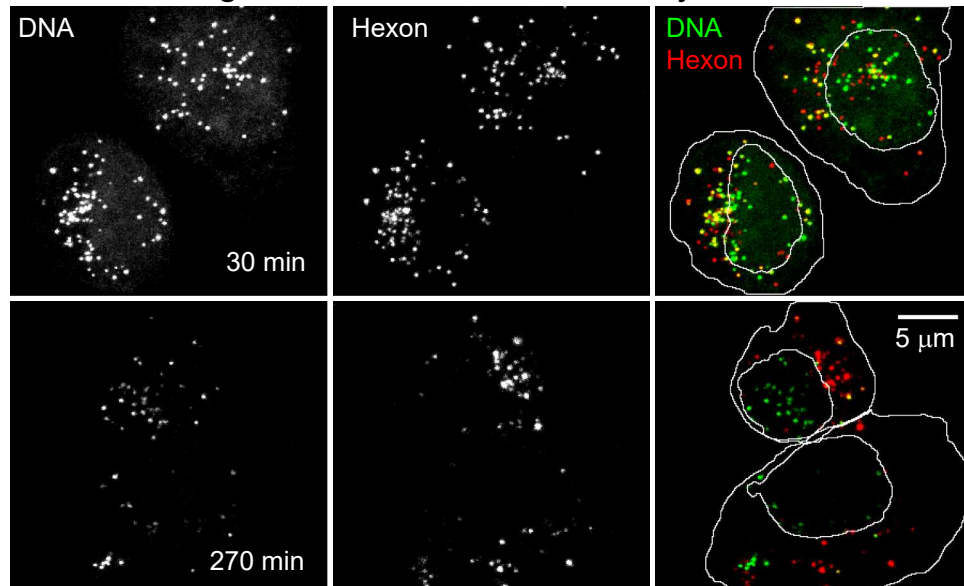

Supplement: S4 Fig — A) Representative images showing protein VI externalization upon virus entry into MPI-2 cells. The DAPI-stained nuclei are shown in blue. Scale bar = 10 μm. B) Representative images for tracking of incoming virus DNA in HAdV-C5-infected MPI-2 cells. The image for the 30 min time point is a maximum projection of a confocal stack through the entire cell volume. Nuclear and cell outlines are indicated. Empty capsid (red) signals in the nuclear area represent capsid remnants below or above the nucleus, whereas the nucleus-associated uncoated DNA (green) can signify either DNA imported into the nucleus, DNA associated with the cytoplasmic side of the nuclear envelope or DNA above or below the nucleus. For the 270 min time point image, confocal slices below and above the nucleus were excluded from the maximum projection, and thus the nucleus-associated uncoated DNA is expected to largely represent DNA imported into the nucleus. Scale bar = 5 μm. (PDF) [file ppat.1006914.s004.pdf]

## S5 Fig

Binding of HAdV-C5, HAdV-B35 and  
HAdV-D26 to MPI-2 cells

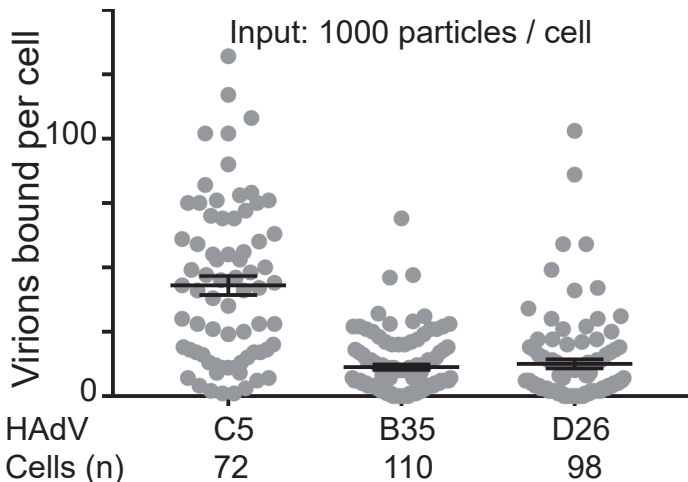

Supplement: S5 Fig — Alexa-Fluor488-labeled viruses (moi ~1000 virus particles per cell) were added to cells at 4°C for 60 min, and after removal of unbound virus, cells were incubated at 37°C for 10 min before fixation. Cells were imaged by confocal microscopy and cell-associated virus particles were scored from maximum projections of confocal stacks. The plot shows number of bound virus particles per cell, one dot representing one cell. Error bars represent the means ± SEMs. Number of cells analyzed is indicated. (PDF) [file ppat.1006914.s005.pdf]

**S6 Fig**

Surface expression of human SR-A6 in transfected HDF-TERT cells

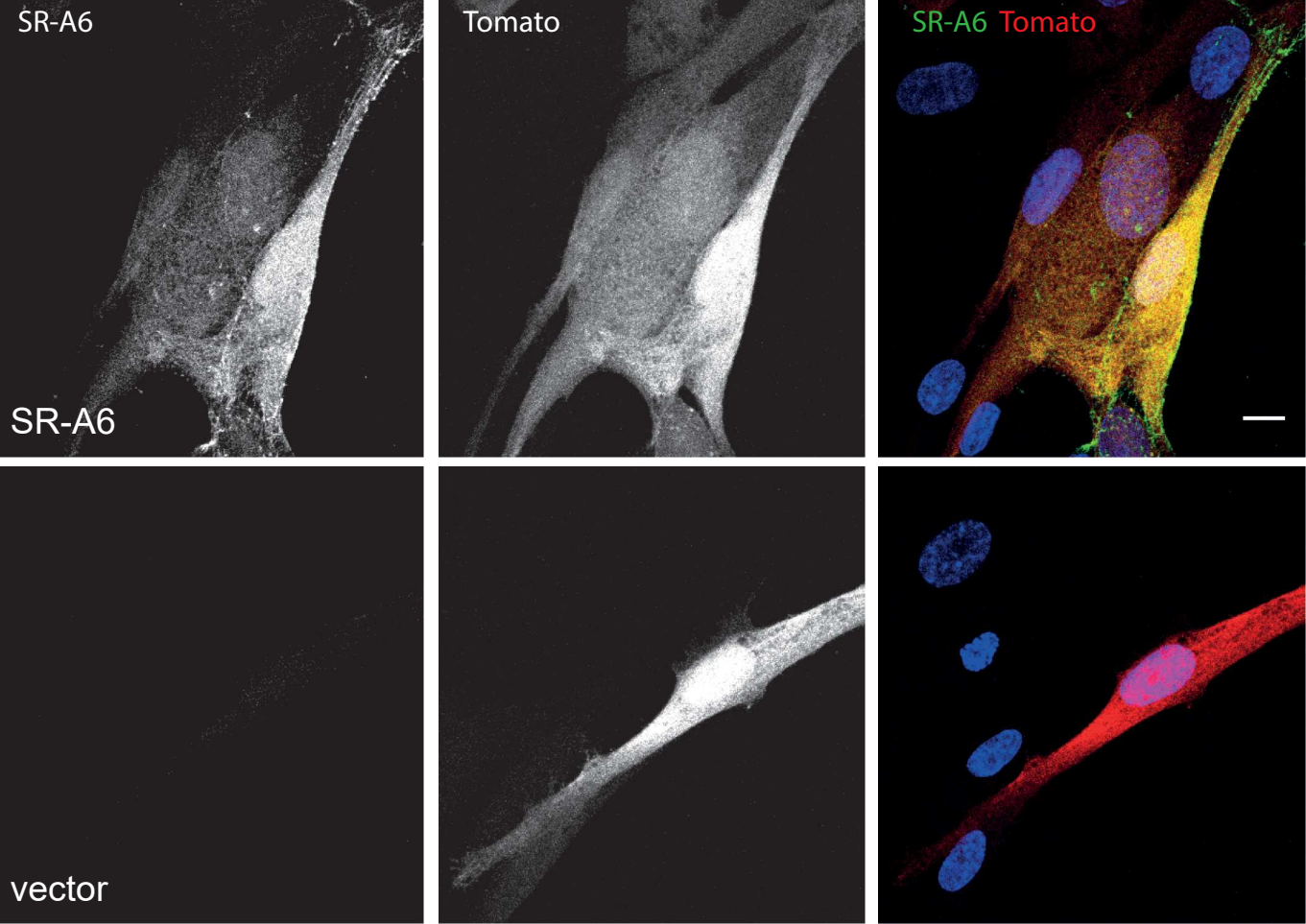

Supplement: S6 Fig — Representative images showing surface expression of human SR-A6 in HDF-TERT cells transfected with a plasmid that directed the synthesis of a bi-cistronic SR-A6-IRES-Tomato mRNA. Control cells were transfected with the empty plasmid backbone. Forty hours post transfection intact cells were incubated with the anti-human SR-A6 PLK1 antibody at 0°C, fixed, incubated with Alexa-Fluor488-conjugated secondary anti-mouse antibodies and DAPI-stained. Images are maximum projections of confocal stacks. Transfected cells displayed variable levels of SR-A6 at the cell surface, and by visual inspection, the intensity of surface SR-A6 signal correlated with the intensity of the Tomato signal. No PLK1 antibody signal was detected on non-transfected Tomato-negative cells or on Tomato-positive cells in the control transfection. Scale bar = 10 μm. (PDF) [file ppat.1006914.s006.pdf]
