# Supplementary material for: Lung macrophage scavenger receptor SR-A6 (MARCO) is an adenovirus type-specific virus entry receptor
Source: PLoS Pathog. 2018 Mar 9;14(3):e1006914. doi: 10.1371/journal.ppat.1006914 (PMC5862501; doi:10.1371/journal.ppat.1006914)
Supplement: S7 Fig — A) Ad5 hexon (PDB ID code 6B1T) is depicted in blue and ribbon corresponding to residues WDEAATALEINLEEEDDDNEDEVDEQAEQQKTHVFGQ, including the HVR1 as defined by [1, 2], is highlighted in red using the molecular visualization program UCSF Chimera. The stretch of amino acids which appears to influence SR-A6 binding is exposed on the surface near the rim of the cup formed in the hexon trimer. B) The HVR1 hexon loops of HAdV-C2 and HAdV-C5 were assigned according to [1], and are indicated in red and acidic residues in green. The sequence alignment was done using Clustal Omega at EMBL-EBI [3]. (PDF) [file ppat.1006914.s007.pdf]

S7 Fig

A HVR1 location on hexon

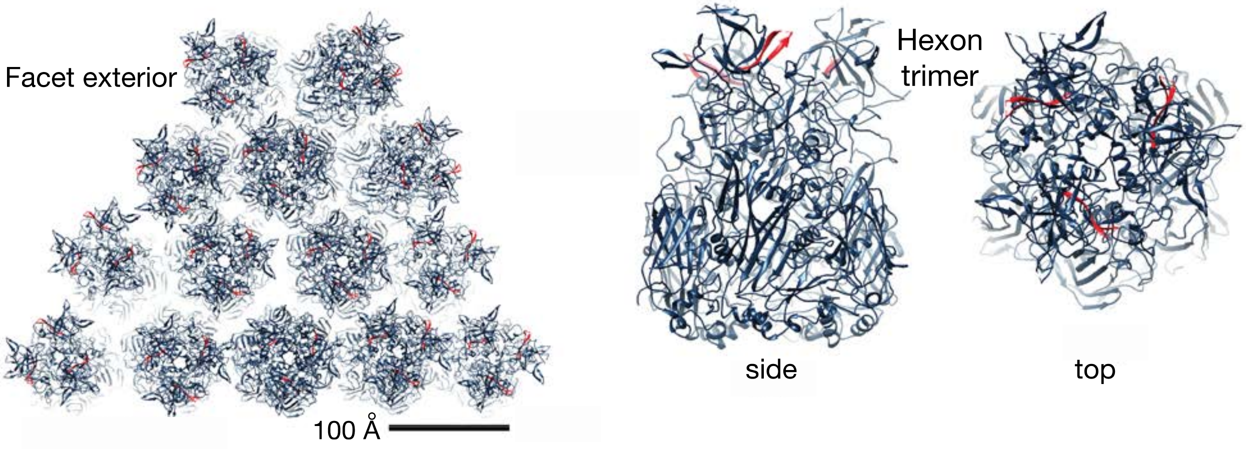

B Alignment of HVR1 sequences

```
                                      HVR1
HAdV C2   YNALAPKGAPNSCEWEQTEDSGRAVAEDEEEEDEDEEEEEEEQNARDQATKKTHVYAQAP
     C5   YNALAPKGAPNPCEWDEAATALEINLEEEDDDNEDEV------------DEQAEQQKTHVFGQAP
     B3   YNSLAPKGAPNTSQWIVTTNGDNAV-----------------------------TTTTNTFGIAS
     B35  YNSLAPKGAPNASQWIAKGVPTAAAAGNGEEE-----------------HETEEKTATYTFANAP
     D26  YNSLAPKGAPNPSQWETKEKQGTTG------------------------GVQQEKDVTKTFGVAA
     A31  YNSLAPKGAPNASQWLTTNNGNK----------------------------------THTFAQAP
```
