# Supplementary material for: Lung macrophage scavenger receptor SR-A6 (MARCO) is an adenovirus type-specific virus entry receptor
Source: PLoS Pathog. 2018 Mar 9;14(3):e1006914. doi: 10.1371/journal.ppat.1006914 (PMC5862501; doi:10.1371/journal.ppat.1006914)
Supplement: S1 Table — (PDF) [file ppat.1006914.s008.pdf]

S2 Table: Oligonucleotides used for qRT-PCR

mSR-A1	5'-TGCTGTCTTCTTTACCAGCA-3'<br>5'-TGAAGGGAGGGGCCATTTTT-3'
mSR-A6	5'-AGGAAGACTTCTTGGGCAGC-3'<br>5'-GAGCAGGATCAGGTGGATGG-3'
mSR-B1	5'- GCCCCAGGTTCTTCACTACG-3'<br>5'- TCCTCAAGAAGCGGGGTGTA-3'
mSR-B2	5'- CAGCTCATACATTGCTGTTTATGC-3'<br>5'- ACTGGTTTTCTCGCCAACTC-3'
mEEF1A1	5'-TCCACTTGGTCGCTTTGCT-3'<br>5'-CTTCTTGTCCACAGCTTTGATGA-3'
mTBP	5'-TTGACCTAAAGACCATTGCACTTC-3'<br>5'-TTCTCATGATGACTGCAGCAAA-3'
mGAPDH	5'- TGGAGTCTACTGGTGTCTTCAC-3'<br>5'- TTTTGGCTCCACCCTTCAAG-3'
