# Supplementary material for: Lung macrophage scavenger receptor SR-A6 (MARCO) is an adenovirus type-specific virus entry receptor
Source: PLoS Pathog. 2018 Mar 9;14(3):e1006914. doi: 10.1371/journal.ppat.1006914 (PMC5862501; doi:10.1371/journal.ppat.1006914)
Supplement: S1 References — (DOCX) [file ppat.1006914.s010.docx]

### Supporting references

1. Rux JJ, Kuser PR, Burnett RM. Structural and phylogenetic analysis of adenovirus hexons by use of high-resolution x-ray crystallographic, molecular modeling, and sequence-based methods. J Virol. 2003;77(17):9553-66. PubMed PMID: 12915569.

2. Dai X, Wu L, Sun R, Zhou ZH. Atomic Structures of Minor Proteins VI and VII in the Human Adenovirus. J Virol. 2017;91(24):e00850-17. Epub 2017/10/06. doi: 10.1128/JVI.00850-17. PubMed PMID: 28978703.

3. Sievers F, Wilm A, Dineen D, Gibson TJ, Karplus K, Li W, et al. Fast, scalable generation of high-quality protein multiple sequence alignments using Clustal Omega. Mol Syst Biol. 2011;7:539. Epub 2011/10/13. doi: 10.1038/msb.2011.75. PubMed PMID: 21988835; PubMed Central PMCID: PMCPMC3261699.
